# Glycoside Hydrolase (GH) 45 and 5 Candidate Cellulases in *Aphelenchoides besseyi* Isolated from Bird’s-Nest Fern

**DOI:** 10.1371/journal.pone.0158663

**Published:** 2016-07-08

**Authors:** Guan-Long Wu, Tzu-Hao Kuo, Tung-Tsuan Tsay, Isheng J. Tsai, Peichen J. Chen

**Affiliations:** 1 Department of Plant Pathology, National Chung Hsing University, Taichung, Taiwan; 2 Biodiversity Research Center, Academia Sinica, Taipei, Taiwan; James Hutton Institute, UNITED KINGDOM

## Abstract

Five *Aphelenchoides besseyi* isolates collected from bird’s-nest ferns or rice possess different parasitic capacities in bird’s-nest fern. Two different glycoside hydrolase (GH) 45 genes were identified in the fern isolates, and only one was found in the rice isolates. A *Abe GH5-1* gene containing an SCP-like family domain was found only in the fern isolates. *Abe GH5-1* gene has five introns suggesting a eukaryotic origin. A maximum likelihood phylogeny revealed that Abe GH5-1 is part of the nematode monophyletic group that can be clearly distinguished from those of other eukaryotic and bacterial GH5 sequences with high bootstrap support values. The fern *A*. *besseyi* isolates were the first parasitic plant nematode found to possess both GH5 and GH45 genes. Surveying the genome of the five *A*. *besseyi* isolates by Southern blotting using an 834 bp probe targeting the GH5 domain suggests the presence of at least two copies in the fern-origin isolates but none in the rice-origin isolates. The *in situ* hybridization shows that the *Abe GH5-1* gene is expressed in the nematode ovary and testis. Our study provides insights into the diversity of GH in isolates of plant parasitic nematodes of different host origins.

## Introduction

The nematode *Aphelenchoides besseyi* is the causal nematode of rice white tip disease, and is one of the most destructive problems in many rice-producing areas[1, 2]. The nematode causes necrosis and whitens the top of rice leaves, eventually causes a drop in the number and size of grains. Large-scale damages resulted from *A*. *besseyi* have been reported around the world. Rice fields with *A*. *besseyi* infection rates of 34–58% were associated to a 35–45% reduction in yield in Taiwan [3], while 60% of rice cultivars in India were found to be infested [4]. Seed samples from 41% of Iranian fields were also infested by *A*. *besseyi* [5]. This nematode has also been found on a wide range of plants, including bird’s-nest fern, strawberry, and sweet corn [2, 6].

Plant parasitic nematodes (PPNs) produce numerous secreted proteins called effectors. The role of some effectors in the interactions between PPNs and their hosts have recently been characterized [7]. The most studied effectors to date are the plant cell wall-degrading enzymes (CWDEs) which have been thought to be acquired from bacteria or fungi via the horizontal gene transfer (HGT) mechanism [7]. CWDEs, which can be produced by both sedentary and migratory PPNs, have been proposed to be required for nematode penetration and migration through plant tissue [8] and for feeding on host cells [9]. Amongst them, genes encoding glycoside hydrolase family 5 (GH5) cellulases have been identified in several nematode species belonging to the *Meloidogyne*, *Globodera*, *Heterodera*, *Pratylenchus*, *Radopholus*, *Ditylenchus*, *Rotylenchulus* and *Aphelenchus* genera [7], of which the majority are obligate plant parasites in the Tylenchoidea superfamily [8]. However, members of the GH45 cellulase family were only found in Parasitaphelenchidae and Aphelenchoididae [10], in which the majority of species are fungivorous and interspersed with several plant-parasitic species (e.g., *Aphelenchoides besseyi*) [11]. The pine wood nematode *Bursaphelenchus xylophilus* contains both GH45 cellulase and GH16 endo-1, 3-β-glucanase [6]. The draft genome assembly of *B*. *xylophilus* did not contain any GH5 domain containing genes [1].

Fu *et al*. (2012) identified three GH5 gene homologues (*Afr-eng-1*, *Afr-eng-2* and *Afr-eng-3*) in *A*. *fragariae*, which is currently the only case of plant parasitic nematodes in the Aphelenchoidea superfamily [8]. The *Afr-eng-1* gene was detected in *A*. *fragariae* collected from hosta plants. When the diet of the *A*. *fragariae* was changed from hosta plants to fungi, fewer individuals in the population were found to bear *Afr-eng-1* [8]. A previous study has revealed that *A*. *besseyi* Taiwanese isolates have different infectivity [12]. In this article, we provide evidence to support coexistence of GH5 and GH45 genes in the genomes of certain *A*. *besseyi* isolates. An additional phylogenetic analysis confirms the *A*. *besseyi* GH5 gene has the common ancestor with other nematode GH5 genes.

## Methods and Materials

### Nematode isolates and their parasitism ability on bird’s-nest fern

The Fm, Fsx and Fgk isolates of *Aphelenchoides besseyi* were collected from bird’s-nest ferns, and the Rl and Rdg isolates were collected from rice. The Fm and Fgk isolates were collected from the wild bird’s-nest fern on the roadside of Mingjian township, Nantou County and Gukeng township, Yunling County, respectively. The Fsx, Rl and Rdg isolates were collected from the cultivated land of private owners when the lab was offering diagnostic service. No specific permissions were required for collecting our research nematode isolates in these locations. Single female isolates of these 5 populations were established according to Jen et al. [13]. Nematodes were reared on *Alternaria citri* that was cultured on a potato dextrose agar slant at 28°C. The identification of Fm, Rl and Rdg was performed according to Hsieh et al. [12]. Single female isolates of Fsx and Fgk were identified based on morphological fine structures such as lateral incisors, tail processes and the length of the post-vulval uterine sac. Using modified primers (S1 Table), partial 18S rDNA sequences (1,667 bp) of all 5 isolates were obtained according to Holterman et al. [14] to confirm their species status (GenBank accession number: Fm, KT454962; Fsx, KT943534; Fgk, KT943535; Rl, KT454963; Rdg, KT943536). The viability and pathogenicity of the three fern-origin isolates were maintained by inoculating the isolates back into their original host plants every 2–3 months.

To test the parasitic ability on bird’s-nest fern, approximately 1,000 all-stage nematodes in 100-μl water suspension were infiltrated into 3-month-old bird’s-nest fern leaves per inoculated site. The *Alternaria citri* hyphal suspension was used as the control. The inoculated plants were kept in a moist chamber for 24 hours. The leaf symptoms were recorded 21 days post inoculation, and nematodes were re-isolated from the inoculation site by modified Baermann funnel method [15].

### Extraction of genomic DNA and RNA from nematodes

Nematodes of all stages were washed with sterile distilled water and cleaned by sucrose flotation before extracting their genomic DNA and RNA. Nematode DNA for PCR experiments was extracted with a Tissue and Cell Genomic DNA Purification kit (GeneMark, Taipei, Taiwan), according to the procedures provided by the manufacturer. An Easy Tissue and Cell Genomic DNA Purification kit (GeneMark, Taipei, Taiwan) was used to extract high quality Nematode DNA for Southern blotting experiment. Total RNA was extracted from *Aphelenchoides besseyi* isolates with an RNeasy Micro kit (Qiagen, Valencia, California, USA), and single-stranded cDNA was synthesized by using SuperScript III Reverse Transcriptase (Invitrogen, California, USA) according to the manufacturer’s instructions.

### Amplification of GH5 and GH45 gene fragments with degenerate primers

The two degenerate primers Eng1, 5′-TAY GTI ATH GTI GAY TGG CA-3′ and Eng2, 5′-GTI CCR TAY TCI GTI ACR AA-3′ [16] were used to amplify nematode GH5 endoglucanases from genomic DNA and cDNA. The two degenerate primers GH45 ENG-1, 5′-ACI MGI TAY TGG GAY TGY TG-3′ and GH45 ENG-2, 5′-RCA ICC RTT RAA IAD ICC IAC-3′ were used to amplify the conserved sequence region of the GH45 cellulase coding gene [10]. PCR reactions were set up in 25 μl of solution containing 5 μl of PCR Plus Master Mix II (GeneMark, Taipei, Taiwan), 1 μl each of 10 μM primer, 2 μl of DNA template and 16 μl of nuclease-free water. The first step in the PCR cycle was denaturation at 94°C for 2 min; followed by 35 cycles at 94°C for 30 s, 47°C for 1 min, and 72°C for 2 min; and a final extension at 72°C for 5 min. The PCR products were separated on a 1.2% agarose gel and stained with ethidium bromide. Potential cellulase gene products were recovered from the gel with a Gel Elution Kit (GeneMark, Taipei, Taiwan, #DP03) and cloned with a TOPO TA Cloning kit (Invitrogen, Carlsbad, California, USA). The successfully transformed bacterial clones were sent to the Mission Biotech Company (Taipei, Taiwan) for sequencing.

### Identification of a full length *Aphenenchoides besseyi* GH45 gene by RACE PCR

A 3’ RACE and 5’ RACE System for the Rapid Amplification of cDNA Ends (Invitrogen, Carlsbad, California, USA, #18373–019 and #18374–058) was used to obtain the 3’ and 5’ cDNA ends of the intended GH45 β -1,4-endoglucanase gene fragment. The following gene-specific primers were used to amplify a GH45 ENG fragment from *A*. *besseyi* Fm and Rl isolates: AbeFm-GH45-GSP-F, AbeRl-GH45-GSP-F, AbeFm-GH45-GSP-R and AbeRl-GH45-GSP-R, AUAP (S1 Table). The PCR reaction was started with a 3 min denaturation at 94°C, followed by 35 cycles of 94°C for 1 min, 55°C for 1 min, and 72°C for 1 min 30 s, and a final extension at 72°C for 5 min.

### Identification of a full length *Aphenenchoides besseyi* GH5 gene by inverse and RACE PCR

Nematode genomic DNA (~50 ng) was digested with *Hinf I* (NEB, California, USA, #Ro155S) and self-ligated with T4 DNA Ligase (GeneMark, Taipei, Taiwan) according to the manufacturer’s protocol. The two inverse primers FmGH5i-F and FmGH5i-R (S1 Table) were designed and synthesized on the basis of the sequence for genomic DNA fragments obtained with the degenerate primers through the LASERGENE^®^ PrimerSelect^™^ program, and the PCR reaction was started with a 3 min denaturation at 94°C, followed by 35 cycles of 94°C for 1 min, 53°C for 1 min, and 72°C for 1 min 30 s, and a final extension at 72°C for 5 min.

The gene-specific primers AbeFm-GH5-GSP-F, AbeFm-GH5-NGSP-F, AbeFm-GH5-GSP-R and AUAP (S1 Table) were used for RACE PCR. The PCR reaction was only different from the iPCR program in terms of the extension temperature of 55°C, which was applied for 1 min.

### Sequence analysis

DNA sequences of 18S used in this study were downloaded from NCBI database [17]. Representative GH5 and GH45 proteins from CAZy database [18] were randomly selected and downloaded from NCBI database. To include GH5 genes from parasitic nematodes with a complete genome, we first used our GH5-1 protein sequence as a query to identify (Blastp [19]; -evalue 1e-3 -max_target_seqs 100000) GH5 orthologues from whole proteomes of plant parasitic nematodes available on Wormbase [20]. The sequences without at least one identified GH5 cellulase (PF00150) domain (less than 1e-10 of pfam_scan.pl version 1.5 [21]; downloaded from ftp://ftp.ebi.ac.uk/pub/databases/Pfam/Tools/) or having length outside 1.5 times of interquartile range were excluded. Redundant sequences were also removed using cdhit [22, 23] with an identity cut off 99%. Additional GH5 protein sequences of nematodes without a complete genome from previous study [24] were also included. The final set of 145 GH5 proteins were classified into subfamilies according to CAZy rules [25].

Sequence alignment data were computed using MAFFT (v7.123b; options—maxiterate 1000—localpair, —maxiterate 1000—localpair and—maxiterate 1000—globalpair for aligning GH5, GH45 and 18S, respectively [26]). The alignment of GH5 and GH45 were trimmed using TrimAL (v1.2 [27]; options -gt 0.05 -w 3 and -gt 0.6 for GH5 and GH45, respectively). Maximum likelihood phylogenies of GH5 and GH45 were computed by FastTree (version 2.1.7 SSE3 [28]) with models of Wag and GAMMA. The maximum likelihood phylogeny of 18S was also computed using FastTree with Generalised time-reversible model. Signal peptide present in GH5 proteins was predicted using SignalP (v4.1 [29]).

### Amplification of *Abe GH5-1*, *Abe GH45-2* and *Abe GH45-3* genes

The primers Abe GH5-1-F, Abe GH5-1-R, Abe GH45-2-F, Abe GH45-2-R, Abe GH45-3-F and Abe GH45-3-R (S1 Table) were designed based on previously known sequences and used to screen for the presence of GH5 and GH45 gene homologues in nematode isolates. The PCR reaction was similar to that of the iPCR program, except that the extension temperature was 50°C for 1 min. The products were visualized and processed for sequencing as previously described.

### Southern blotting and *in situ* hybridization of the *Abe GH5-1* gene

Approximately 10 to 20 μg of genomic DNA from the 5 different *A*. *besseyi* isolates was digested with *Hinf I* (NEB, California, USA) overnight at 37°C. The digested DNA was separated on a 0.8% agarose gel and blotted onto a positively charged nylon membrane (GE Healthcare Biosciences). A DNA probe was amplified and labelled with digoxigenin-11-dUTP (Roche, Mannheim, Germany) using primers Abe GH5-1-S-F and Abe GH5-1-S-R (S1 Table). Southern blot hybridization was performed at 55°C overnight, and the probe was detected with a DIG Luminescent Detection Kit (Roche, Mannheim, Germany, #11363514910) according to the manufacturer’s instructions and CCD image detector ChemiDoc MP with Image Lab^™^ (Bio-Rad Laboratories, Inc., CA, USA) was used to acquire the image.

An *in situ* hybridization procedure was performed by following the protocol described by de Boer *et al*. [30] with some modifications. Mixed stages of nematodes were digested by proteinase K for 30 minutes to break down the cuticle without cutting with the razor blade. A digoxigenin-labelled *A*. *besseyi Abe GH5-1* RNA probe was synthesized following the protocol of the Roche DIG RNA Labelling kit (Roche, Mannheim, Germany, #11175025910). The sense and anti-sense probes were obtained by *in vitro* transcription and labelled with digoxigenin-11-UTP with Abe GH5-1-ISH-F, Abe GH5-1-ISH-R, T7- Abe GH5-1-ISH-F and T7- Abe GH5-1-ISH-R primers (S1 Table). The nematodes were transferred onto the slides and examined microscopically (Olympus BX50, Germany).

## Results

### Characterization of five *Aphelenchoides besseyi* isolates from rice and fern host origins

**Fig 1:**
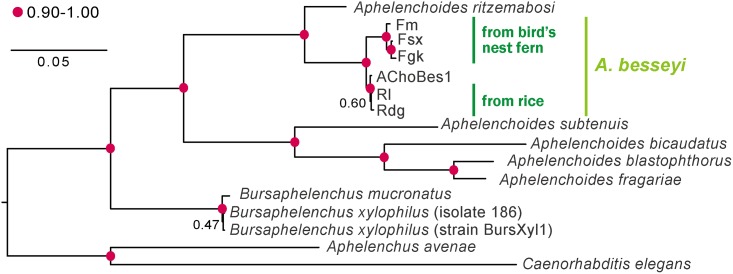
A maximum likelihood phylogeny of 18S DNA sequences. The phylogeny shows that *Aphelenchoides besseyi* are clustered together, and our sequences from different host plants can also be distinguished. The bootstrap values are indicated by numbers of percentage near each node, and a value higher than 90 are presented using red dots. GenBank ids of sequences include: EU196001.1 (*Caenorhabditis elegans*), AY508034.1 (*Bursaphelenchus xylophilus* isolate 186), KJ636306.1 (*Bursaphelenchus xylophilus* strain BursXyl1), AY284648.1 (*Bursaphelenchus mucronatus*), JQ348399.1 (*Aphelenchus avenae*), JQ957879 (*Aphelenchoides blastophthorus*), AY284643.1 (*Aphelenchoides bicaudatus*), JQ957890.1 (*Aphelenchoides subtenuis*), JQ957881.1 (*Aphelenchoides ritzemabosi*), AJ966475.1 (*Aphelenchoides fragariae*), and JQ957878.1 (*Aphelenchoides besseyi* isolate AChoBes1).

*A*. *besseyi* isolates originating from bird’s-nest fern (Fm, Fgk, Fsx) and those originating from rice (Rl, Rdg) were used in this study. The isolates Fm, Rl and Rdg were identified to species level in the previous study [12]. Both Fsx and Fgk isolates had an off-set lip region, metacorpus greater than 75% body width, three to four pointed processes on the tail and four lateral incisures when observed under the light microscope, and the post-vulval uterine sac was about 2.5 times of the anal body width long (S1 Fig). These morphological characteristics were sufficient to support the species level identification [31]. Maximum-likelihood phylogeny deduced from the 18S sequences of the 5 isolates and outgroup species placed the *A*. *besseyi* isolates into the same group, with isolates from different origins clearly separated (Fig 1).

**Fig 2:**
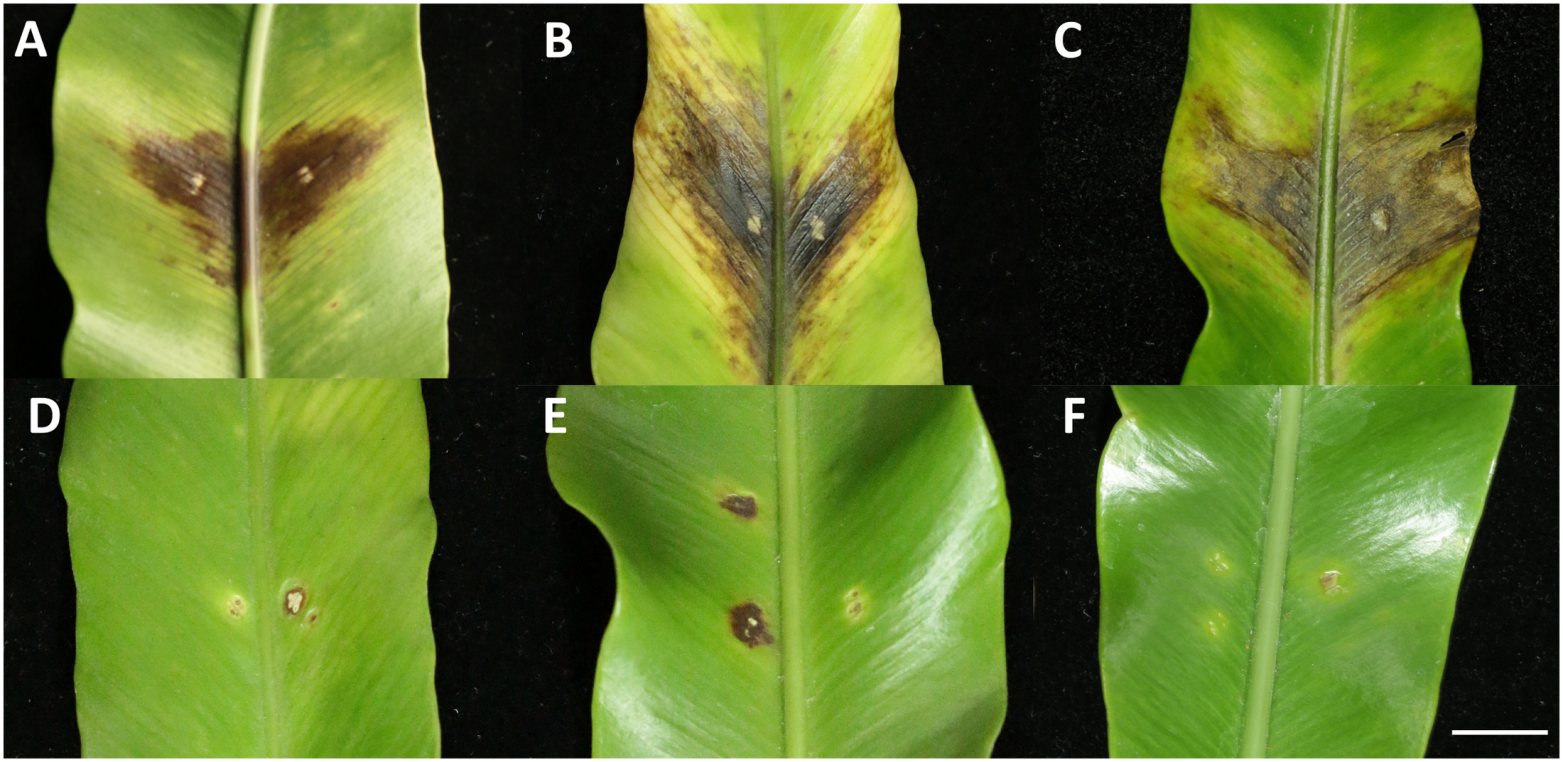
The symptoms on the bird’s-nest fern leaves 21 days after inoculated with different host-origin *Aphelenchoides besseyi* isolates. The bird’s-nest fern leaves inoculated with the (A) Fm, (B) Fsx and (C) Fgk isolates showed typical dark-brown patches. No symptoms were observed on bird’s-nest fern leaves inoculated with the rice-origin (D) Rl and (E) Rdg isolates. Leaves treated with *Alternaria citr*i hyphal suspensions were used as (F) controls. The scale bars represent 10 cm.

Twenty-one days after inoculation, only the bird’s-nest fern-origin *A*. *besseyi* isolates could cause the typical dark-brown patch symptoms on the bird’s-nest fern (Fig 2A–2C) and the *A*. *besseyi* nematodes were re-isolated from the symptomatic leaves. The rice-origin Rl and Rdg isolates did not cause visible symptoms (Fig 2D and 2E) on the fern leaves and no nematodes were found from the inoculation site, by either staining or isolation.

### Identification of GH family genes in *Aphelenchoides besseyi*

**Fig 3:**
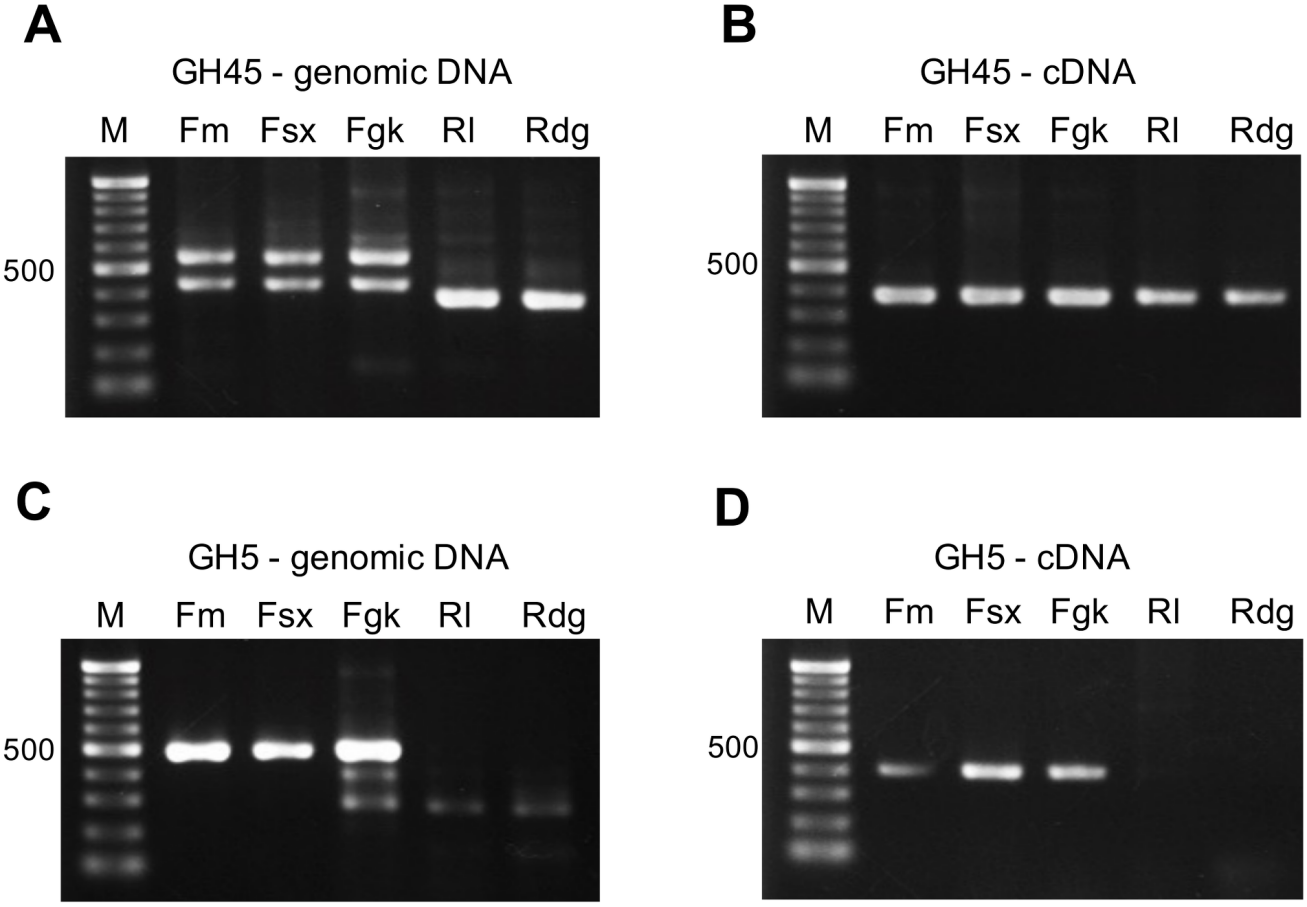
Amplified profiles of three bird’s-nest fern-origin isolates (Fm, Fsx and Fgk) and two rice-origin isolates (Rl and Rdg) of *Aphelenchoides besseyi* genomic DNA and cDNA with the GH45 and GH5 degenerate primer sets. The GH 45 degenerate primers amplified two major bands of 548 and 439 bp (A) from Fm, Fsx and Fgk isolates and a 387 bp band from genomic DNA of the Rl and Rdg isolates. (B) A single band of 387 bp was amplified from cDNA of all test isolates. The GH5 degenerate primers amplified a major 491 bp band (C) from genomic DNA and (D) a 407 bp band from the cDNA of the Fm, Fsx and Fgk isolates.

We sought to identify putative glycoside hydrolases (GH) in our five isolates of *A*. *besseyi* by amplification with various degenerate primers set corresponding to different GH family genes. Using the GH45 degenerate primers, three different gene fragments were amplified from the genomic DNA of all five *A*. *besseyi* isolates (Fig 3A). The bird’s-nest fern-origin isolates yielded two major bands of 548 and 439 bp, while a single 387-bp band was amplified from the two rice-origin isolates. The minor bands were not reproducible during experiments. Two cDNA fragments of 390 bp and 393 bp, were sequenced from the three bird’s-nest fern-origin isolates and the two rice-origin isolates could only find a 387-bp cDNA fragment (Fig 3B). Using the GH5 degenerate primers, a major 491-bp band was amplified from the genomic DNA of all fern-origin isolates. However, the Fgk isolate had another 2 minor bands of 407 and 238-bp. The gDNA of the rice-origin isolates Rl and Rdg also had the 238-bp band (Fig 3C). Sequences from the two minor bands showed no homology to any known genes in NCBI nr database and so were therefore considered to be miss amplification. A single 407-bp band was amplified from the cDNA of the three *A*. *besseyi* Fm, Fsx, and Fgk isolates, but not from the Rl and Rdg isolates (Fig 3D).

### GH45 genes present in the *Aphelenchoides besseyi* Fm and Rl isolates

**Fig 4:**
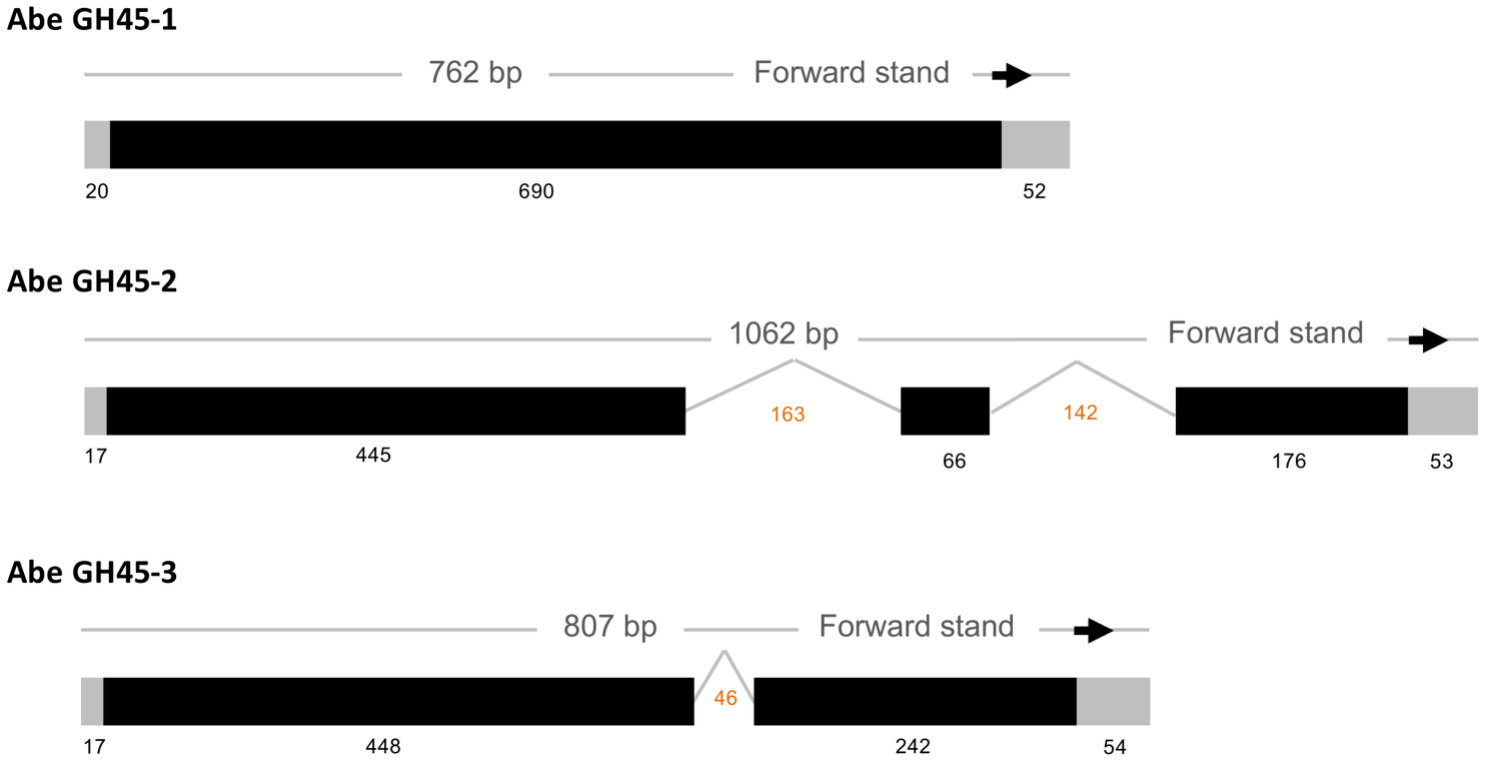
The gene structures of three GH45 genes in *Aphelenchoides besseyi*. The introns not to exist in the *Abe GH45-1* and presence in the *Abe GH45-2* and *Abe GH45-3* genes. Two introns, 163 bp and 142 bp, were found in the *Abe GH45-2* and a 46-bp sized intron was found in the *Abe GH45-3* gene.

**Fig 5:**
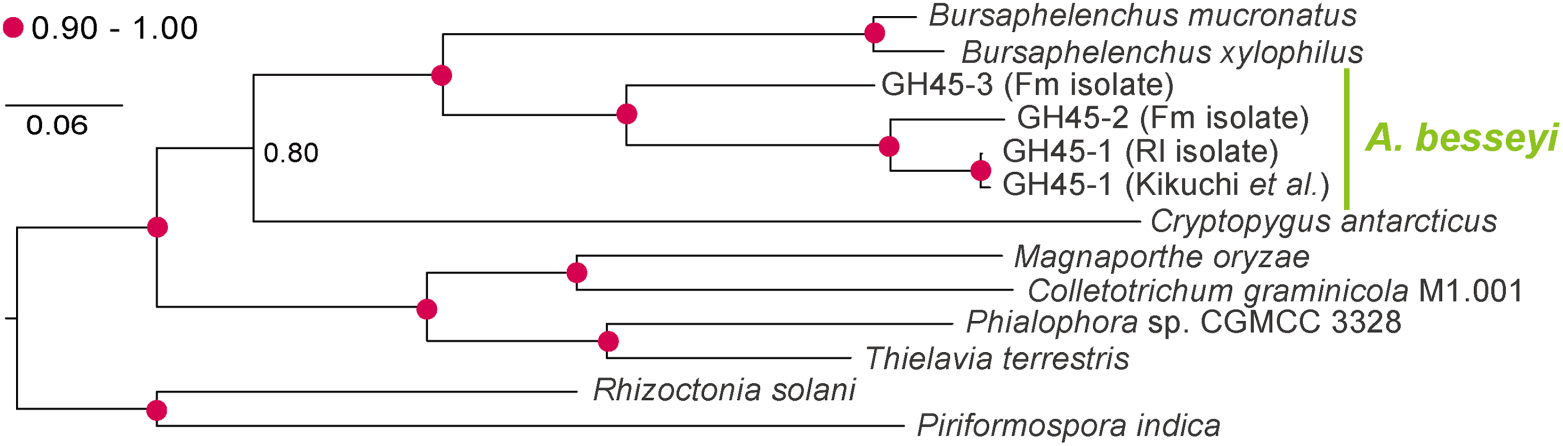
A maximum likelihood phylogeny of GH45 orthologues. The bootstrap support values are shown as percentage values near nodes, in which those higher than 90 are presented using red dots. GenBank ids of sequences are described here: CCA72362.1 (*Piriformospora indica*), EHA54445.1 (*Magnaporthe oryzae*), ADV02790.1 (*Rhizoctonia solani*), ADZ99360.1 (*Phialophora* sp. CGMCC 3328), AEO65930.1 (*Thielavia terrestris*), ACV50414.1 (*Cryptopygus antarcticus*), AFD53063.1 (*Bursaphelenchus mucronatus*), ACD12136.1 (*Bursaphelenchus xylophilus*), and XP_008089512.1 (*Colletotrichum graminicola* M1.001).

To determine the full sequences of GH45 in populations of *A*. *besseyi* from fern and rice, the cDNA of the *A*. *besseyi* Fm and Rl isolates amplified by the degenerate primers were used for RACE PCR. A 229 amino acid open reading frame was identified from the 777-bp cDNA fragment that was amplified from the Rl isolate of *A*. *besseyi* (NCBI #KP754666), which was designated as *Abe GH45-1*. Two cDNA fragments were amplified from the Fm isolate, which encode 228 and 229 aa (NCBI #KP754667 and KP754668) polypeptides and were designated *Abe GH45-2* and *Abe GH45-3*, respectively. Moreover, the sequences amplified from Fm and Rl genomic DNA showed that two introns are present in *Abe GH45-2* and one in *Abe GH45-3*, but is absent in the *Abe GH45-1* (Fig 4). Each protein predicted from the three DNA sequences contains a GH45 cellulase domain. Our alignment data of the protein sequences also revealed conserved domains responsible for cellulase activity (S2 Fig), in consistent with findings in a published study of Wang *et al* [6]. A phylogeny based on all possible GH45 proteins in nematodes and representative outgroup sequences placed Abe GH45-1 and Abe GH45-2 in one group along with GH45 from a Japanese *A*. *besseyi* isolate collected from rice [1] (Fig 5). Interestingly, the second copy of GH45 gene (Abe GH45-3) in the Fm isolate was placed outside the group and displayed only 78% identity with the Abe GH45-1 protein.

### Identification of a novel GH5 gene in the *Aphelenchoides besseyi* Fm isolate

**Fig 6:**
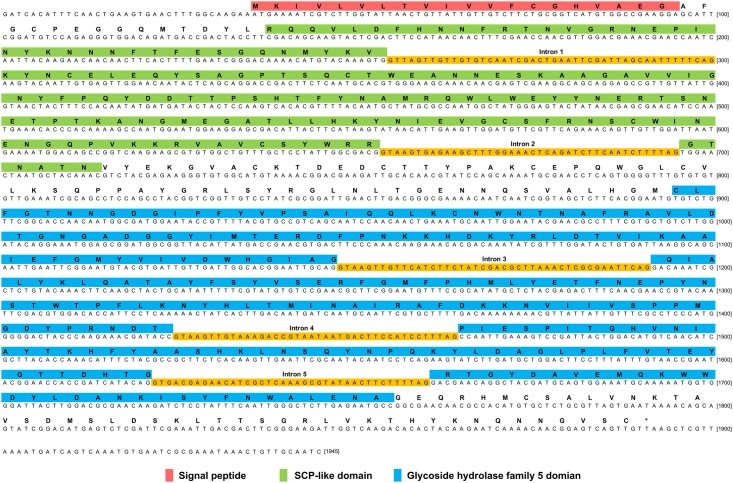
The protein and genomic DNA sequences of *Abe GH5-1*. The Abe GH5-1 protein contains a SCP-like domain at the N-terminus and a GH5 cellulase domain at the C-terminus, as well as five introns were found in the *Abe GH5-1* gene.

A 1,748 bp putative GH5 mRNA sequence was assembled by inverse and RACE PCR and ended with a 14 poly-A tail from the Fm isolate, which we designated *Abe GH5-1*. The ORF start codon was predicted by the ORF finder and consisted of a 1647 bp ORF sequence before the stop codon. Conceptually translated amino acids (548 aa, from NCBI #KM035416) contain a signal peptide (1–20 aa) and two different domains: the expected GH5 cellulase (258–503 aa) domain, and a sperm-coating protein (SCP)-like extracellular protein superfamily domain (35–197 aa) at its N-terminus (Figs 6 and S3 and S4). Comparison of genomic and cDNA sequences revealed the presence of five introns (Fig 6), suggesting that this gene originates from eukaryotes excluding the possibility of bacterial contamination from a close association with *A*. *besseyi*.

**Fig 7:**
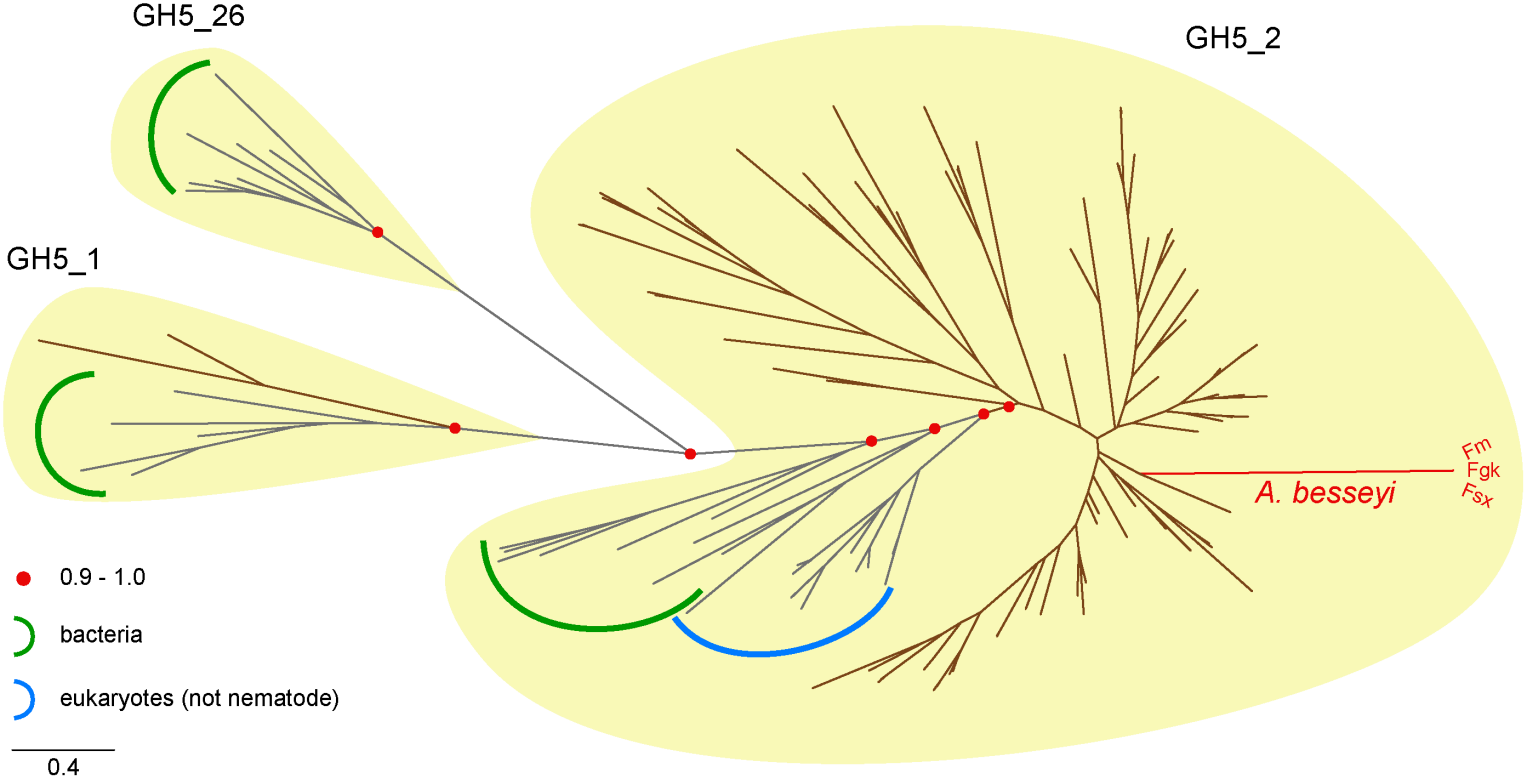
A maximum likelihood phylogeny of nematode GH5 proteins. Orthologues of nematodes are represented using brown branches, and those of other species are shown as grey branches labeled with either green (bacteria) or blue (non-nematode eukaryotes) arcs. Nematodes included in GH5_1 are *Pristionchus pacificus* (clade V). The phylogeny was computed using FastTree (as described in the Materials and Methods), and a high bootstrap support values of 0.91 reveals that GH5 orthologues of nematodes can be clearly distinguished from those of other eukaryotes and bacteria. For more details, please see S5 Fig.

We sought to investigate the evolutionary scenario of the two domains found in Abe GH5-1. First, classification based on CAZy database [25] placed Abe GH5-1 in subfamily GH5_2, currently the largest in family GH5. We conducted a maximum likelihood phylogeny of all GH5 sequences of nematodes and representative sequences within GH5_2 and different GH5 subfamilies. The phylogeny shows clear separation of different subfamilies of GH5 (Figs 7 and S5). Within GH5_2, sequences from clade IV nematodes, including Abe GH5-1, form a monophyletic group with a bootstrap support value 0.91. This phylogeny suggests the distinction within GH5 orthologues of nematode, non-nematode eukaryotes and bacteria in the GH5_2 subfamily. The nematode-origin GH5 sequence that was classified in the GH5_1 subfamily was from *Pristionchus pacificus* suggesting independent lateral gene transfer events to clade IV nematodes [32]. Second, a maximum likelihood phylogeny with SCP sequences from representative platyhelminths placed the orthologues of *A*. *besseyi* in the monophyletic nematode group of SCP major subfamily 1 (S6 Fig; [33]).

### Presence and absence of *GH* gene in five *Aphelenchoides besseyi* isolates

**Fig 8:**
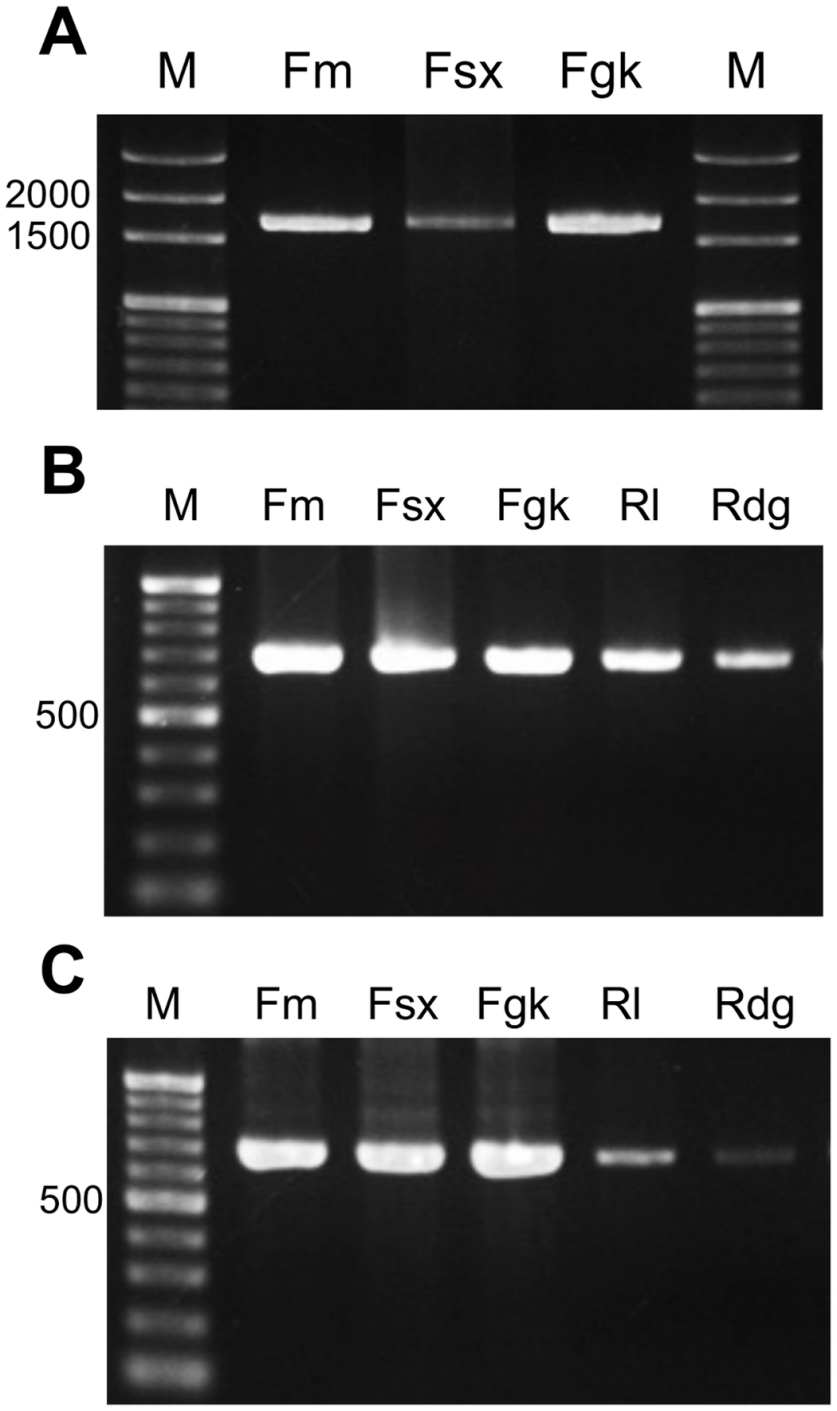
The amplification profiles of *Aphelenchoides besseyi* isolates; (A) *Abe GH5-1* gene, (B) *Abe GH45-2* and (C) *Abe GH45-3*. (A) An *Abe GH5-1* gene product of 1688 bp was amplified exclusively from the Fm, Fsx and Fgk isolates. Both *Abe GH45-2* (B) and *Abe GH45-3* (C) primers could amplify a major band from all five *Aphelenchoides besseyi* isolates.

A 1688-bp fragment showing similarity with the GH5 gene was amplified from three bird’s-nest fern-origin isolates with the *Abe GH5-1* gene-specific primers (Fig 8A), and the sequences were 100% identical to *Abe GH5-1* (NCBI #KM035416). The primer sets for *Abe GH45-2* and *Abe GH45-3* could amplify all the *A*. *besseyi*-isolate cDNA. However, the sequences of these PCR products showed that the primers were only specific to Fm, Fsx and Fgk isolates. The bands amplified by these 2 sets of primers from the 2 rice-origin isolates Rl and Rdg were *Abe GH45-1* (Fig 8B and 8C).

### Southern blotting and *in situ* hybridization of the *Abe GH5-1* gene in *Aphelenchoides besseyi* Fm isolate

**Fig 9:**
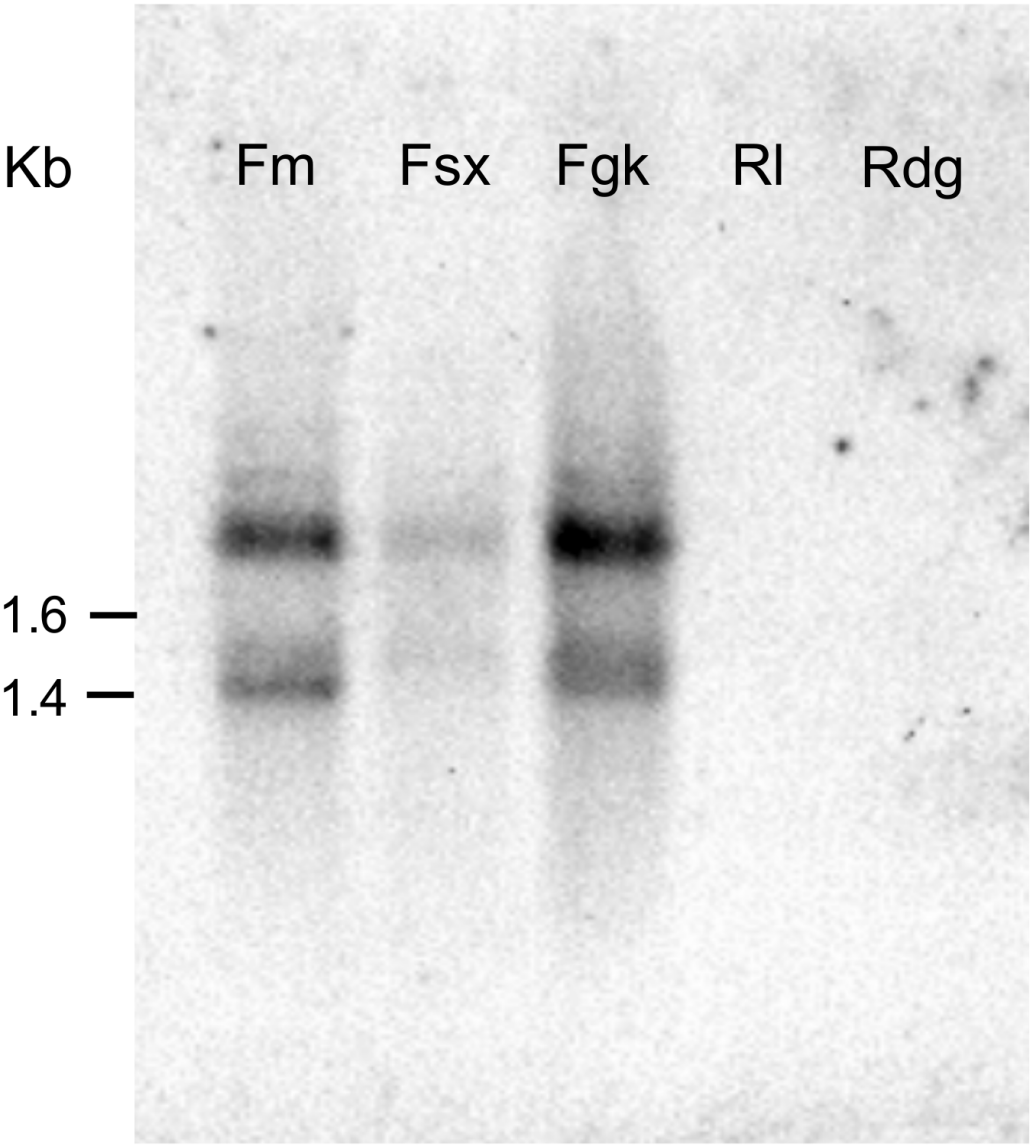
Identification of the *Abe GH5-1* gene by Southern blotting. Genomic DNA isolated from the five *Aphelenchoides besseyi* isolates were digested with *Hinf I*, and it was hybridized with an AbeFm-GH5-specific DNA probe.

An 834 bp DNA probe containing partial GH5 domain with two introns was amplified with Abe GH5-1-S-F and Abe GH5-1-S-R primers from genomic DNA of the *A*. *besseyi* Fm isolate and used for Southern blot analyses. The probe hybridized to two major bands from nematode genomic DNA samples that had been digested by *Hinf I* enzymes (Fig 9), indicating the presence of two copies of the partial GH5 domains of *Abe GH5-1* gene in the three bird’s-nest fern-origin isolates and absence in the two rice-origin isolates.

**Fig 10:**
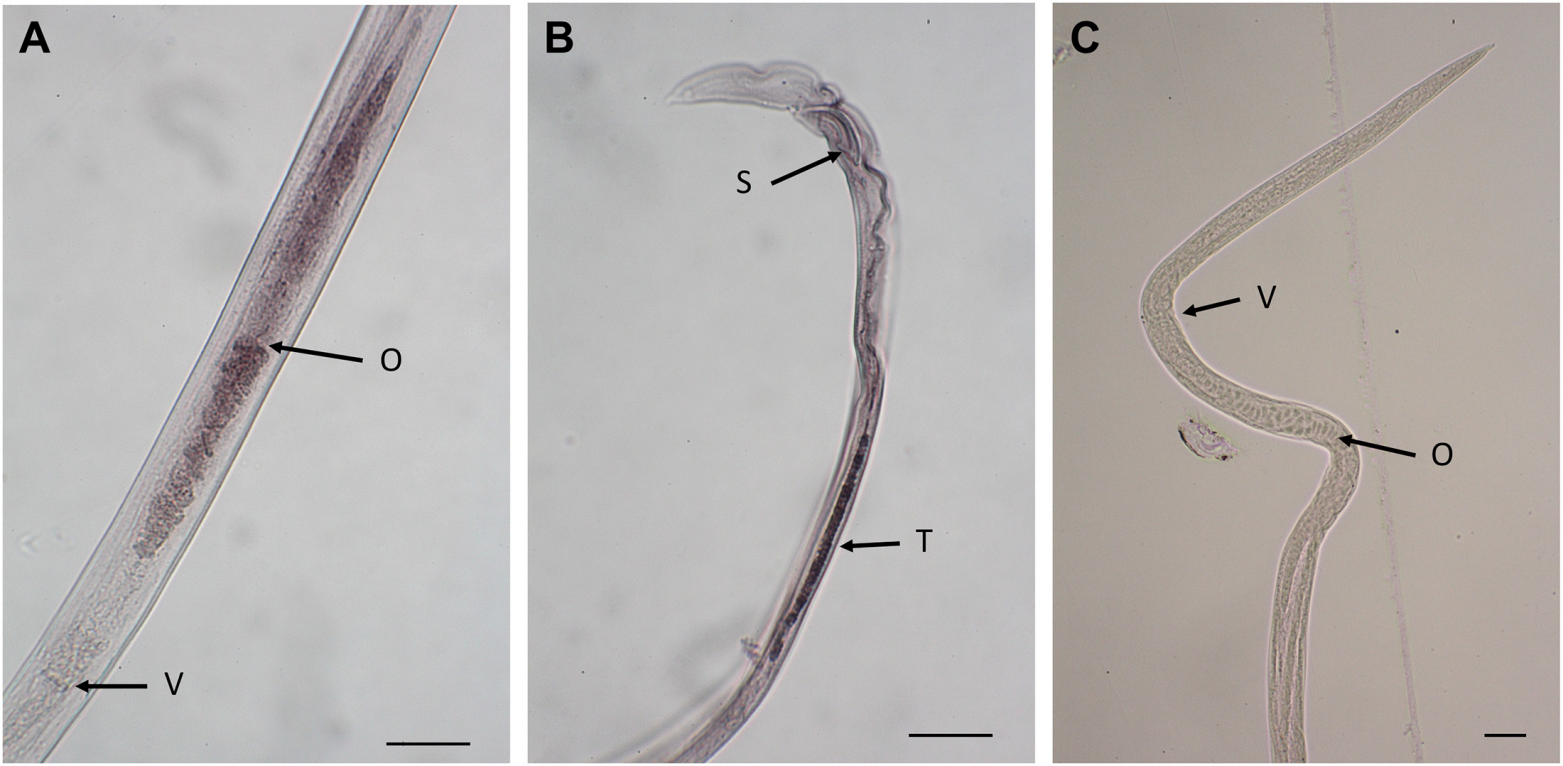
Localization of the *Abe GH5-1* gene transcript in the *Aphelenchoides besseyi* by *in situ* hybridization with digoxigenin-labelled antisense (A and B) or sense probes (C). The scale bars represent 20 μm. O: ovary, V: vulva, S: spicule and T: testis.

The approximately 1,300 bp sense and antisense probes containing entire GH5 domain and partial SCP-like domain without any introns of the *Abe GH5-1* gene were synthesized for *in situ* hybridization. The anti-sense hybridization was highlighted on the reproductive tracts of both female and male *A*. *besseyi* Fm isolate (Fig 10A and 10B), and no mRNA was detected by the sense probe (Fig 10C). Interestingly, *Abe GH5-1* genes were grouped with *O*. *ostertagi* SCP members that were highly expressed and localized in reproductive tract but distant to the third member that was localized in esophagus (S6 Fig; [34]).

## Discussion

Our observation on morphometric traits and the computational analysis of 18S phylogeny show that our five isolates are *A*. *besseyi*. Consistent with their different ability to parasitize on bird’s-nest ferns, isolates of rice and bird’s nest fern are clearly separated into two groups suggesting some fundamental genomic differences between different host-origin isolates. There are huge variations in numbers and types of glycoside hydrolase in plant parasitic nematodes [35]. This study is the first report on the co-existence of both candidate GH5 and GH45 cellulase-coding genes in a single PPN species. We have identified a candidate GH5 and two GH45 cellulase genes in all three *A*. *besseyi* isolates that were originally collected from the bird’s-nest fern but only one GH45 cellulase-coding gene was currently identified from the two rice-origin isolates.

The protein identities of the two GH45 cellulases found in bird’s-nest fern-origin isolates were 90% and 78% compared with the rice-origin isolate described by Kikuchi *et al*. (2014) [1] (S2 Fig). Three *Golobodera tabacum* subspecies with different host ranges were found to have polymorphisms on three effector genes which were CWDEs [36]. The differences of the GH45 gene from the different host-origin *A*. *besseyi* isolates also correlated with their differences in parasitic capabilities [37]. An analysis of offspring from crosses between bird’s-nest ferns and rice-origin isolates revealed that some of the offspring acquired the capability to parasitize bird’s-nest ferns from the paternal isolate, implying a shift in the effects of morbific genes [12]. Whether the differences of the CWDEs in these *A*. *besseyi* isolates attribute to their parasitic capabilities or simply the accumulated variations from divergence still awaits study.

SCP-like domain has been found in a wide range of species with high levels of divergence between related species. The SCP/TAPS genes in parasites are believed to play different roles in the host-pathogen interactions [38]. Although most discovered members in the helminths SCP/TAPS family contain a single SCP domain [39], novel combinations of SCP are beginning to emerge with the influx of nematode genomes [40, 41]. Genes with both SCP and bacterial origin domains have been previously reported [41], but function of these genes remains to be elucidated. As a first step, our SCP-domain containing *Abe GH5-1* gene was expressed and localised in ovary and testis (Fig 10A and 10B). In our SCP phylogeny (S6 Fig), Abe GH5-1 is closely grouped with *Ostertagia ostertagi* ASP members localised in reproductive tracts (bootstrap value 0.9). A large repertoire of SCP/TAPS proteins have been upregulated in sex-specific stages or cells across various species and have been implicated in mediating cell interactions between reproductive physiology [42, 43]. Moreover, the SCP domain is located at N-terminus suggesting that at least the *Abe GH5-1* localisation signal may be ancestral to SCP origin.

GH5 cellulases are commonly found in the Tylenchida plant parasitic nematodes. There are three types of catalytic domains that can be distinguished among those GH5 cellulases [24], and previous study suggested that these GH5 genes were passed on by ancestors of a family nowadays known as the Pratylenchidae. In the superfamily Aphelenchoidea, GH5 were found present in *Aphelenchus avenae* [24], *A*. *fragariae* [8] and in *A*. *besseyi*, but not *B*. *xylophilus* [35]. Our GH5 phylogeny is consistent to previous conclusion that one or more multiple gene duplications took place since the acquisition of a GH5 gene from a common nematode ancestor (S5 Fig; [24]), and Abe-GH5-1 is placed in the catalytic domain- type B group [24]. In this revised phylogeny with more complete genomes, sub families consisting different species are observed again consistent with multiple gene duplication in the early Pratylenchidae common ancestor. Interestingly, the two *A*. *fragariae* GH5 proteins are grouped with *Ditylenchus spp*. but not with Abe-GH5-1. There are a few explanations to this. First, gene fusion is a fundamental evolutionary mechanism for gaining of functions [44]. As Abe-GH5-1 is a product of fusion of GH5 and SCP/TAPS domain containing genes, it may be under different selection scenario [45] to other nematode GH5 genes leading to differential substitution rate. Second, there is a high possibility that there will be multiple GH5 copies present in *A*. *besseyi*, as evident in published parasitic nematode genomes [46, 47]. An *A*. *besseyi* GH5 as a result of multiple duplication from the acquired copy that is more closely related to the two *A*. *fragariae* GH5 proteins may be present. Alternatively, different GH5s may be present in the Aphelenchoides ancestor and then differentially lost in *A*. *besseyi* and *A*. *fragariae*. More complete nematode genomes in basal Tylenchida will help further delineating the relationship of GH5 in this position of the phylogeny. Nevertheless, the real function of Abe GH5-1 warrants further investigation.

The HGT of CWDEs in PPNs is a critical factor that drives nematodes from free-living to plant parasitism [48–50]. The genes acquired via HGT could be integrated into any linkage of the nematode chromosomes. It was interesting to investigate how nematodes retain and activate the HGT genes, despite the obvious divergence in transcriptional and translational mechanisms between donor and receiver species, resulting in the inability to express most of the transferred genes [49]. The *Abe GH5-1* genes were found only in bird’s-nest fern-origin *A*. *besseyi* isolates and not in the rice-origin ones that were not able to parasitize ferns, strongly implying that the environment or host may play an important role in whether nematodes retain the HGT genes during evolution.

## Supporting Information

S1 Fig Photomicrographs of female *Aphelenchoides besseyi* Fsx (A-D) and Fgk (E-H) isolates. (PDF)

S2 Fig Alignment of predicted GH45 amino acid sequences from *Aphelenchoides besseyi* Abe GH45-1, Abe GH45-2 and Abe GH45-3 with GH45 proteins from other *A*. *besseyi*. (PDF)

S3 Fig SignalP output of the Abe GH5-1 protein sequence. According to the score distribution, only one signal peptide can be detected in the Abe GH5-1 protein.
(PDF)

S4 Fig Hypothetical proteins translated from the partial *Abe GH5-1* cDNA sequence. (PDF)

S5 Fig Full phylogeny of GH5 orthologues. (PDF)

S6 Fig A maximum likelihood phylogeny of SCP proteins. (PDF)

S1 Table Primers used to amplify *Aphelenchoides besseyi* cDNA and genomic DNA. (PDF)
