# Supplementary material for: Glycoside Hydrolase (GH) 45 and 5 Candidate Cellulases in Aphelenchoides besseyi Isolated from Bird’s-Nest Fern
Source: PLoS One. 2016 Jul 8;11(7):e0158663. doi: 10.1371/journal.pone.0158663 (PMC4938546; doi:10.1371/journal.pone.0158663)
Supplement: S1 Fig — (PDF) [file pone.0158663.s001.pdf]

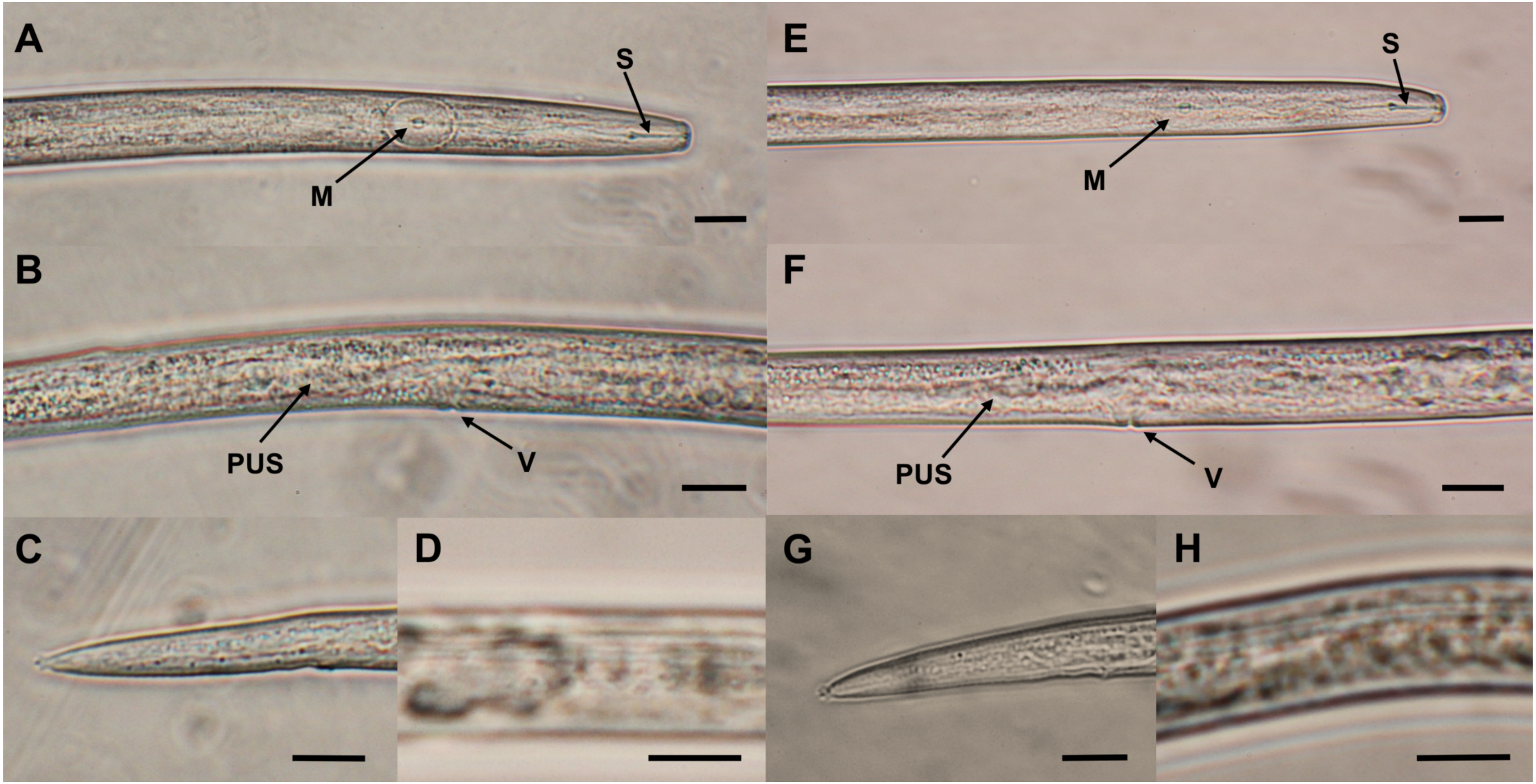

S1 Fig. Photomicrographs of female *Aphelenchoides besseyi* Fsx (A-D) and Fgk (E-H) isolates. (A, E) Anterior region of adult females; S indicates stylet and M indicates middle bulb. (B, F) Posterior region; PUS = post-vulval uterine sac. and V indicates vulva. (C, G) Tail is star-shape with 3-4 pointed processes. (D, H) Lateral field with 4 incisures. The scale bars represent 10 µm.
