# Supplementary material for: Glycoside Hydrolase (GH) 45 and 5 Candidate Cellulases in Aphelenchoides besseyi Isolated from Bird’s-Nest Fern
Source: PLoS One. 2016 Jul 8;11(7):e0158663. doi: 10.1371/journal.pone.0158663 (PMC4938546; doi:10.1371/journal.pone.0158663)
Supplement: S2 Fig — (PDF) [file pone.0158663.s002.pdf]

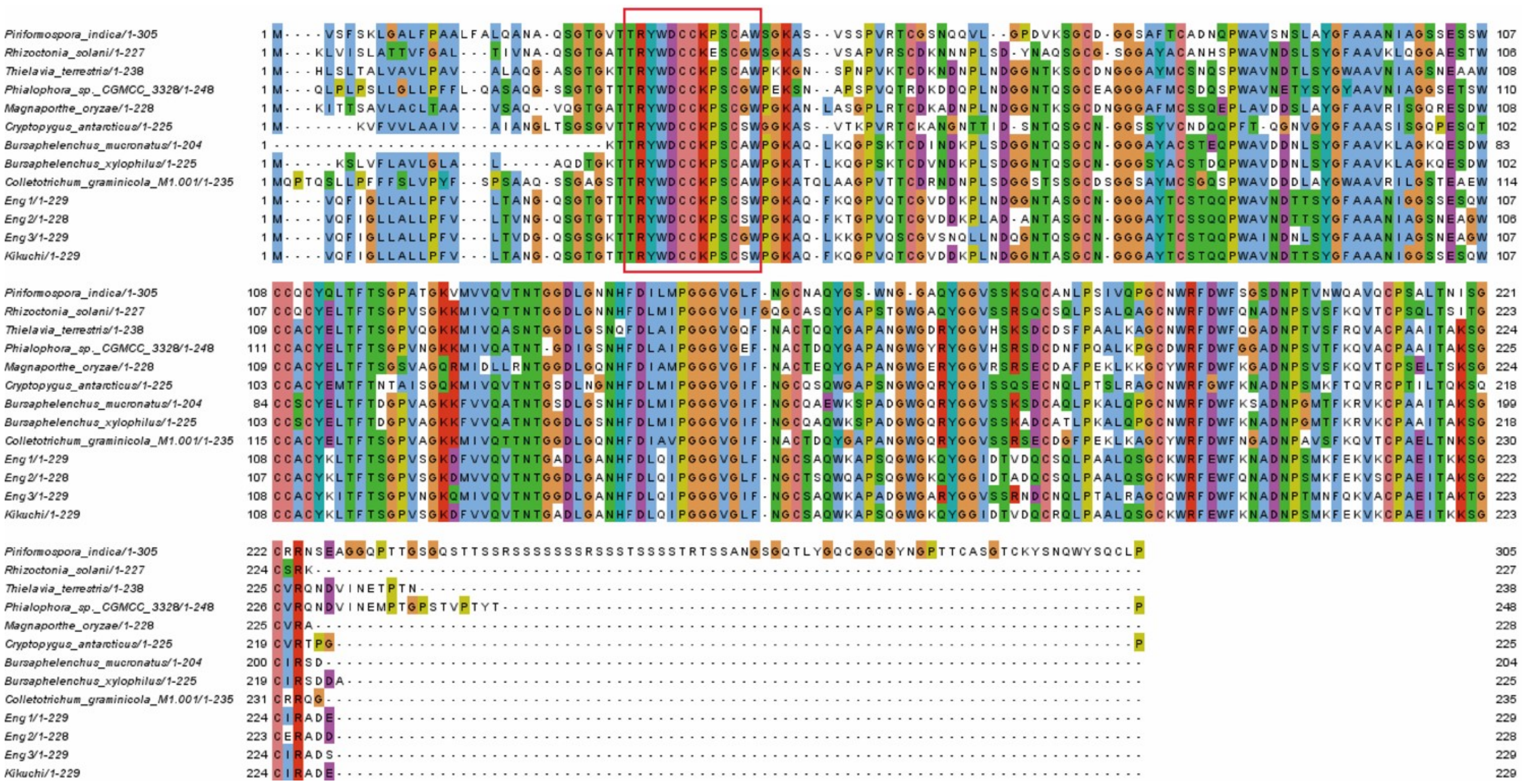

S2 Fig. Alignment of predicted GH45 amino acid sequences from *Aphelenchoides besseyi* Abe GH45-1, Abe GH45-2 and Abe GH45-3 with GH45 proteins from other *A. besseyi* [1].

Reference:

1. Kikuchi T, Helder J, Cock PJA, Jones JT. Characterisation of the transcriptome of Aphelenchoides besseyi and identification of a GHF 45 cellulase. Nematology. 2014;16(1):99-107. doi: 10.1163/15685411-00002748.
