# Supplementary material for: Glycoside Hydrolase (GH) 45 and 5 Candidate Cellulases in Aphelenchoides besseyi Isolated from Bird’s-Nest Fern
Source: PLoS One. 2016 Jul 8;11(7):e0158663. doi: 10.1371/journal.pone.0158663 (PMC4938546; doi:10.1371/journal.pone.0158663)
Supplement: S3 Fig — According to the score distribution, only one signal peptide can be detected in the Abe GH5-1 protein. (PDF) [file pone.0158663.s003.pdf]

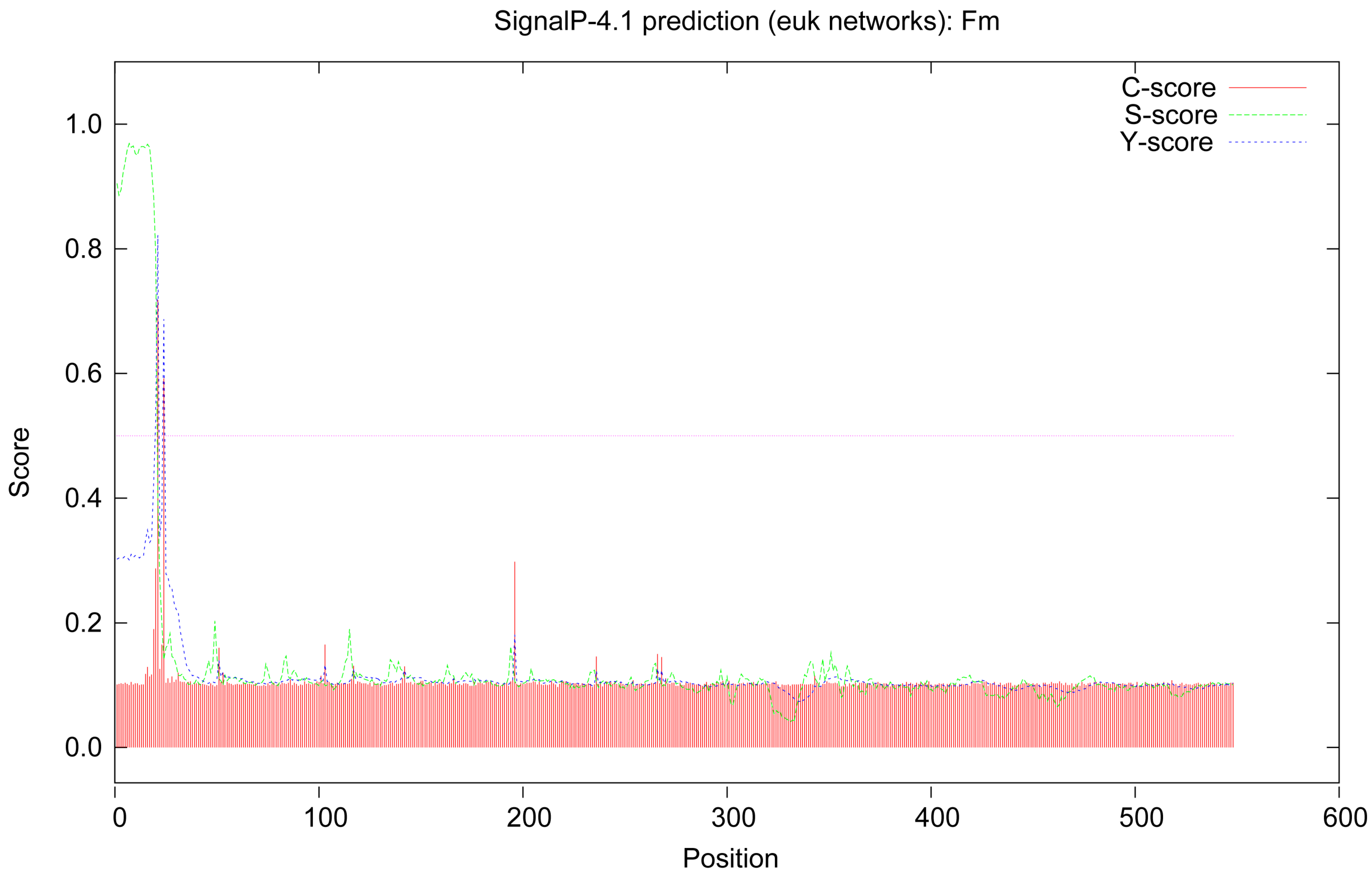

S3 Fig. SignalP output of the Abe GH5-1 protein sequence. According to the score distribution, only one signal peptide can be detected in the Abe GH5-1 protein.
