# Supplementary material for: Glycoside Hydrolase (GH) 45 and 5 Candidate Cellulases in Aphelenchoides besseyi Isolated from Bird’s-Nest Fern
Source: PLoS One. 2016 Jul 8;11(7):e0158663. doi: 10.1371/journal.pone.0158663 (PMC4938546; doi:10.1371/journal.pone.0158663)
Supplement: S4 Fig — (PDF) [file pone.0158663.s004.pdf]

```
     W  N  *  C  Y  K  R  L  R  E  G  C  G  M  *  N  G  R  R  L     F1
      G  T  N  A  T  N  V  Y  E  K  G  V  A  C  K  T  D  E  D  C    F2
       E  L  M  L  Q  T  S  T  R  R  V  W  H  V  K  R  T  K  I  A   F3
   1 TGGAACTAATGCTACAAACGTCTACGAGAAGGGTGTGGCATGTAAAACGGACGAAGATTG 60
     ----:----|----:----|----:----|----:----|----:----|----:----|

     H  N  V  S  S  K  M  R  T  S  V  G  F  V  C  V  E  I  A  A     F1
      T  T  Y  P  A  K  C  E  P  Q  W  G  L  C  V  L  K  S  Q  P    F2
       Q  R  I  Q  Q  N  A  N  L  S  G  V  C  V  C  *  N  R  S  L   F3
  61 CACAACGTATCCAGCAAAATGCGAACCTCAGTGGGGTTTGTGTGTGTTGAAATCGCAGCC 120
     ----:----|----:----|----:----|----:----|----:----|----:----|

     S  S  L  R  S  V  V  L  S  R  I  E  L  D  G  R  K  Q  S  I     F1
      P  A  Y  G  R  L  S  Y  R  G  L  N  L  T  G  E  N  N  Q  S    F2
       Q  P  T  V  G  C  P  I  A  D  *  T  *  R  A  K  T  I  N  R   F3
 121 TCCAGCCTACGGTCGGTTGTCCTATCGCGGATTGAACTTGACGGGCGAAAACAATCAATC 180
     ----:----|----:----|----:----|----:----|----:----|----:----|

     G  S  S  S  R  N  V  S  V  R  H  Q  Q  W  R  W  N  T  V  L     F1
      V  A  L  H  G  M  C  L  F  G  T  N  N  G  D  G  I  P  F  Y    F2
       *  L  F  T  E  C  V  C  S  A  P  T  M  A  M  E  Y  R  F  T   F3
 181 GGTAGCTCTTCACGGAATGTGTCTGTTCGGCACCAACAATGGCGATGGAATACCGTTTTA 240
     ----:----|----:----|----:----|----:----|----:----|----:----|

     R  A  V  S  N  P  T  T  E  M  Q  L  E  Y  E  R  L  S  C  C     F1
      V  P  S  A  I  Q  Q  L  K  C  N  W  N  T  N  A  F  R  A  V    F2
       C  R  Q  Q  S  N  N  *  N  A  I  G  I  R  T  P  F  V  L  S   F3
 241 CGTGCCGTCAGCAATCCAACAACTGAAATGCAATTGGAATACGAACGCCTTTCGTGCTGT 300
     ----:----|----:----|----:----|----:----|----:----|----:----|

     L  G  Y  R  K  W  S  G  W  R  L  H  Y  D  R  T  *  L  P  K     F1
      L  D  T  G  N  G  A  D  G  G  Y  I  M  T  E  R  D  F  P  N    F2
       W  I  Q  E  M  E  R  M  A  V  T  L  *  P  N  V  T  S  Q  T   F3
 301 CTTGGATACAGGAAATGGAGCGGATGGCGGTTACATTATGACCGAACGTGACTTCCCAAA 360
     ----:----|----:----|----:----|----:----|----:----|----:----|

     Q  E  T  R  Q  I  S  F  G  Y  C  D  *  G  S  N  *  I  R  N     F1
      K  K  H  D  K  Y  R  L  D  T  V  I  K  A  A  I  E  F  G  M    F2
       R  N  T  T  N  I  V  W  I  L  *  L  R  Q  Q  L  N  S  E  C   F3
 361 CAAGAAACACGACAAATATCGTTTGGATACTGTGATTAAGGCAGCAATTGAATTCGGAAT 420
     ----:----|----:----|----:----|----:----|----:----|----:----|

     V  R  D  C  *  L  A  R  N  C  R  T  N  R  S  V  Q  T  S  S     F1
      Y  V  I  V  D  W  H  G  I  A  G  Q  I  A  L  Y  K  L  Q  A    F2
       T  *  L  L  I  G  T  E  L  Q  D  K  S  L  C  T  N  F  K  L   F3
 421 GTACGTGATTGTTGATTGGCACGGAATTGCAGGACAAATCGCTCTGTACAAACTTCAAGC 480
     ----:----|----:----|----:----|----:----|----:----|----:----|

     Y  C  I  F  F  V  C  V  R  T  L  R  N  V  S  A  Y  A  L  R     F1
      T  A  Y  F  S  Y  V  S  E  R  F  G  M  F  P  H  M  L  Y  E    F2
       L  H  I  F  R  M  C  P  N  A  S  E  C  F  R  I  C  S  T  R   F3
 481 TACTGCATATTTTTCGTATGTGTCCGAACGCTTCGGAATGTTTCCGCATATGCTCTACGA 540
     ----:----|----:----|----:----|----:----|----:----|----:----|

     D  F  Q  R  T  V  Q  F  D  V  D  T  I  P  Q  K  L  S  L  D     F1
      T  F  N  E  P  Y  N  S  T  W  T  P  F  L  K  N  Y  H  L  T    F2
       L  S  T  N  R  T  I  R  R  G  H  H  S  S  K  T  I  T  *  Q   F3
 541 GACTTTCAACGAACCGTACAATTCGACGTGGACACCATTCCTCAAAAACTATCACTTGAC 600
     ----:----|----:----|----:----|----:----|----:----|----:----|

     N  D  Q  C  N  S  C  F  *  Q  K  K  R  Y  Y  C  F  A  S  H     F1
      M  I  N  A  I  R  A  F  D  K  K  N  V  I  I  V  S  P  P  M    F2
       *  S  M  Q  F  V  L  L  T  K  K  T  L  L  L  F  R  L  P  W   F3
 601 AATGATCAATGCAATTCGTGCTTTTGACAAAAAAAACGTTATTATTGTTTCGCCTCCCAT 660
     ----:----|----:----|----:----|----:----|----:----|----:----|

     G  G  L  P  K  K  R  Y  P  N  *  K  S  D  Y  W  T  C  Q  H     F1
      G  D  Y  P  R  N  D  T  P  I  E  S  P  I  T  G  H  V  N  I    F2
       G  T  T  Q  E  T  I  P  Q  L  K  V  R  L  L  D  M  S  T  S   F3
 661 GGGGGACTACCCAAGAAACGATACCCCAATTGAAAGTCCGATTACTGGACATGTCAACAT 720
     ----:----|----:----|----:----|----:----|----:----|----:----|

     R  L  H  Q  T  F  L  R  R  F  S  Q  V  E  F  A  I  Q  S  S     F1
      A  Y  T  K  H  F  Y  A  A  S  H  K  L  N  S  Q  Y  N  P  Q    F2
       L  T  P  N  I  S  T  P  L  L  T  S  *  I  R  N  T  I  L  R   F3
 721 CGCTTACACCAAACATTTCTACGCCGCTTCTCACAAGTTGAATTCGCAATACAATCCTCA 780
     ----:----|----:----|----:----|----:----|----:----|----:----|

     E  V  S  *  C  W  T  S  F  I  C  N  R  I  R  N  H  R  S  Y     F1
      K  Y  L  D  A  G  L  P  L  F  V  T  E  Y  G  T  T  D  H  T    F2
       S  I  L  M  L  D  F  L  Y  L  *  P  N  T  E  P  P  I  I  Q   F3
 781 GAAGTATCTTGATGCTGGACTTCCTTTATTTGTAACCGAATACGGAACCACCGATCATAC 840
     ----:----|----:----|----:----|----:----|----:----|----:----|

     R  T  N  R  L  R  C  S  G  N  A  K  M  V  G  L  L  G  R  E     F1
      G  R  T  G  Y  D  A  V  E  M  Q  K  W  W  D  Y  L  D  A  N    F2
       D  E  Q  A  T  M  Q  W  K  C  K  N  G  G  I  T  W  T  R  T   F3
 841 AGGACGAACAGGCTACGATGCAGTGGAAATGCAAAAATGGTGGGATTACTTGGACGCGAA 900
     ----:----|----:----|----:----|----:----|----:----|----:----|

     Q  D  L  L  F  Q  L  G  S  *  E  C  R  R  T  T  P  H  V  L     F1
      K  I  S  Y  F  N  W  A  L  E  N  A  G  E  Q  R  H  M  C  S    F2
       R  S  P  I  S  I  G  L  L  R  M  P  A  N  N  A  T  C  A  L   F3
 901 CAAGATCTCCTATTTCAATTGGGCTCTTGAGAATGCCGGCGAACAACGCCACATGTGCTC 960
     ----:----|----:----|----:----|----:----|----:----|----:----|

     C  V  S  E  *  N  S  S  I  G  H  E  S  R  F  E  I  D  D  F     F1
      A  L  V  N  K  T  A  V  S  D  M  S  L  D  S  K  L  T  T  S    F2
       R  *  *  I  K  Q  Q  Y  R  T  *  V  S  I  R  N  *  R  L  R   F3
 961 TGCGTTAGTGAATAAAACAGCAGTATCGGACATGAGTCTCGATTCGAAATTGACGACTTC 1020
     ----:----|----:----|----:----|----:----|----:----|----:----|

     G  K  I  G  Q  D  T  L  Q  E  S  K  Q  R  S  Q  L  L  S  S     F1
      G  R  L  V  K  T  H  Y  K  N  Q  N  N  G  V  S  C  *  A  R    F2
       E  D  W  S  R  H  T  T  R  I  K  T  T  E  S  V  V  K  L  V   F3
1021 GGGAAGATTGGTCAAGACACACTACAAGAATCAAAACAACGGAGTCAGTTGTTAAGCTCG 1080
     ----:----|----:----|----:----|----:----|----:----|----:----|

     L  K  *  S  V                                                  F1
      *  N  D  Q  S                                                 F2
       K  M  I  S  X                                                F3
1081 TTAAAATGATCAGTC 1095
     ----:----|----:
```

pfam domain PF00150

signal peptide

putative peptides in frame 1

putative peptides in frame 2

putative peptides in frame 3

S4 Fig. Hypothetical proteins translated from the partial *Abe GH5-1* cDNA sequence. To understand whether the Abe GH5-1 protein can be produced without SCP-like domain, we computationally generated incomplete proteins by excluding a 5' region of two exons from the GH5 cDNA sequence, which covered the coding region of signal peptide and SCP-like domain. Using the website tool EMBOSS sixpack [1], the incomplete DNA sequence was translated into protein sequences with different open reading frames. Signal peptides and pfam domains of the proteins were detected using SignalP (v4.1 [2]) and pfam_scan.pl (version 1.5 [3]). Our data show that no single protein containing both signal peptide and GH5 domain can be translated from our *Abe GH5-1* cDNA sequence once the 5' region was not available.

Reference:

1. Rice P, Longden I, Bleasby A. EMBOSS: the European molecular biology open software suite. Trends in genetics. 2000;16(6):276-7.

2. Petersen TN, Brunak S, von Heijne G, Nielsen H. SignalP 4.0: discriminating signal peptides from transmembrane regions. Nat Methods. 2011;8(10):785-6. doi: 10.1038/nmeth.1701. PubMed PMID: 21959131.

3. Finn RD, Bateman A, Clements J, Coggill P, Eberhardt RY, Eddy SR, et al. Pfam: the protein families database. Nucleic acids research. 2013:gkt1223.
