# Supplementary material for: Glycoside Hydrolase (GH) 45 and 5 Candidate Cellulases in Aphelenchoides besseyi Isolated from Bird’s-Nest Fern
Source: PLoS One. 2016 Jul 8;11(7):e0158663. doi: 10.1371/journal.pone.0158663 (PMC4938546; doi:10.1371/journal.pone.0158663)
Supplement: S5 Fig — (PDF) [file pone.0158663.s005.pdf]

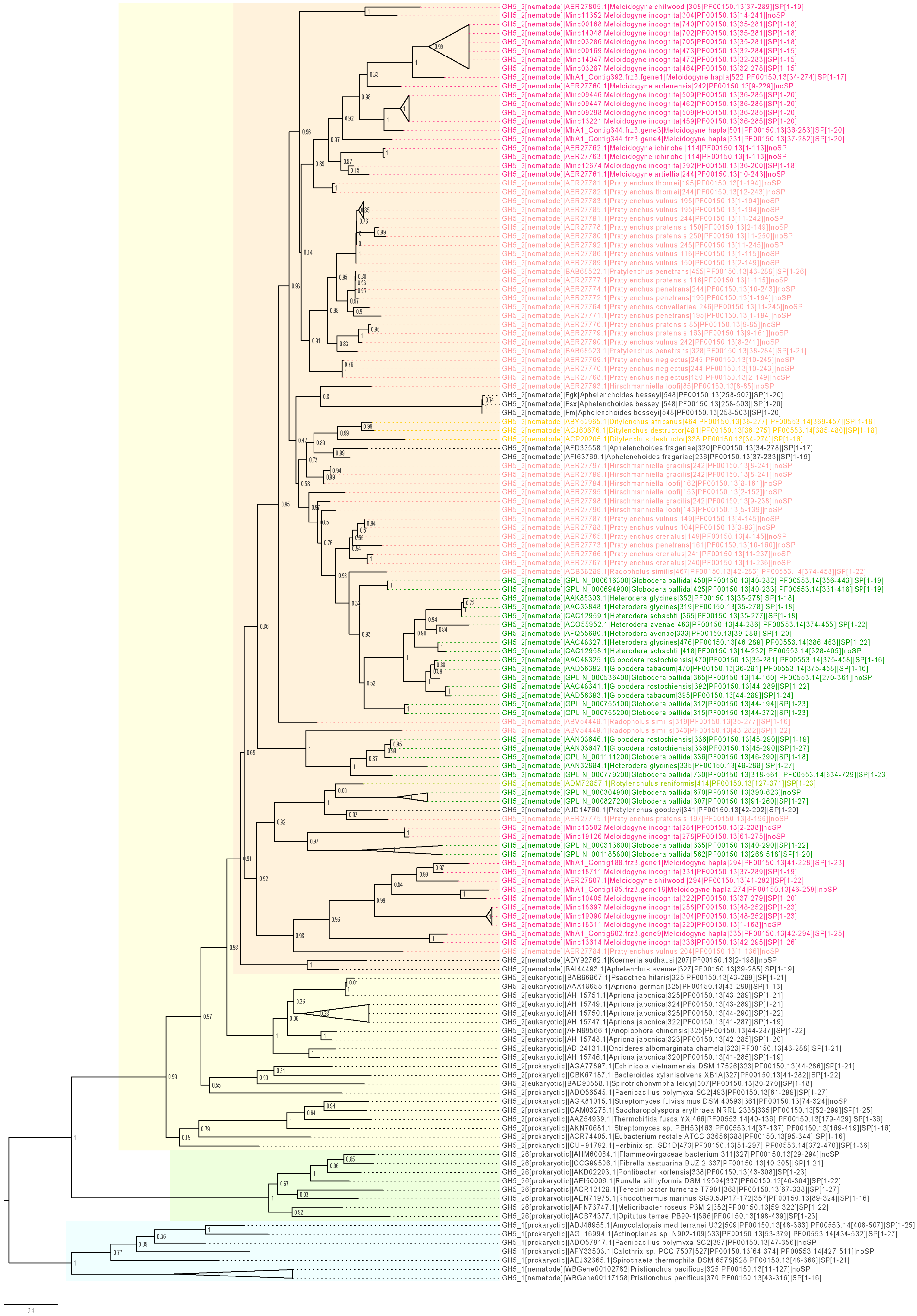
GH5_2[nematode]|AER27805.1|Meloidogyne chitwoodi|308|PF00150.13[37-289]|SP[1-19]
GH5_2[nematode]|Minc11352|Meloidogyne incognita|304|PF00150.13[14-241]|noSP
GH5_2[nematode]|Minc00168|Meloidogyne incognita|740|PF00150.13[35-281]|SP[1-18]
GH5_2[nematode]|Minc14048|Meloidogyne incognita|702|PF00150.13[35-281]|SP[1-18]
GH5_2[nematode]|Minc03286|Meloidogyne incognita|705|PF00150.13[35-281]|SP[1-18]
GH5_2[nematode]|Minc00169|Meloidogyne incognita|473|PF00150.13[32-284]|SP[1-15]
GH5_2[nematode]|Minc14047|Meloidogyne incognita|472|PF00150.13[32-283]|SP[1-15]
GH5_2[nematode]|Minc03287|Meloidogyne incognita|464|PF00150.13[32-278]|SP[1-15]
GH5_2[nematode]|MhA1_Contig392.frz3.fgene1|Meloidogyne hapla|522|PF00150.13[34-274]|SP[1-17]
GH5_2[nematode]|AER27760.1|Meloidogyne ardenensis|242|PF00150.13[9-229]|noSP
GH5_2[nematode]|Minc09446|Meloidogyne incognita|509|PF00150.13[36-285]|SP[1-20]
GH5_2[nematode]|Minc09447|Meloidogyne incognita|462|PF00150.13[36-285]|SP[1-20]
GH5_2[nematode]|Minc09298|Meloidogyne incognita|509|PF00150.13[36-285]|SP[1-20]
GH5_2[nematode]|Minc13221|Meloidogyne incognita|459|PF00150.13[36-285]|SP[1-20]
GH5_2[nematode]|MhA1_Contig344.frz3.gene3|Meloidogyne hapla|501|PF00150.13[36-283]|SP[1-20]
GH5_2[nematode]|MhA1_Contig344.frz3.gene4|Meloidogyne hapla|331|PF00150.13[37-282]|SP[1-20]
GH5_2[nematode]|AER27762.1|Meloidogyne ichinohei|114|PF00150.13[1-113]|noSP
GH5_2[nematode]|AER27763.1|Meloidogyne ichinohei|114|PF00150.13[1-113]|noSP
GH5_2[nematode]|Minc12674|Meloidogyne incognita|292|PF00150.13[36-200]|SP[1-18]
GH5_2[nematode]|AER27761.1|Meloidogyne artiellia|244|PF00150.13[10-243]|noSP
GH5_2[nematode]|AER27781.1|Pratylenchus thornei|195|PF00150.13[1-194]|noSP
GH5_2[nematode]|AER27782.1|Pratylenchus thornei|244|PF00150.13[12-243]|noSP
GH5_2[nematode]|AER27783.1|Pratylenchus vulnus|195|PF00150.13[1-194]|noSP
GH5_2[nematode]|AER27785.1|Pratylenchus vulnus|195|PF00150.13[1-194]|noSP
GH5_2[nematode]|AER27791.1|Pratylenchus vulnus|244|PF00150.13[11-242]|noSP
GH5_2[nematode]|AER27778.1|Pratylenchus pratensis|150|PF00150.13[2-149]|noSP
GH5_2[nematode]|AER27780.1|Pratylenchus pratensis|250|PF00150.13[11-250]|noSP
GH5_2[nematode]|AER27792.1|Pratylenchus vulnus|245|PF00150.13[11-245]|noSP
GH5_2[nematode]|AER27786.1|Pratylenchus vulnus|116|PF00150.13[1-115]|noSP
GH5_2[nematode]|AER27789.1|Pratylenchus vulnus|150|PF00150.13[2-149]|noSP
GH5_2[nematode]|BAB68522.1|Pratylenchus penetrans|455|PF00150.13[43-288]|SP[1-26]
GH5_2[nematode]|AER27777.1|Pratylenchus pratensis|116|PF00150.13[1-115]|noSP
GH5_2[nematode]|AER27774.1|Pratylenchus penetrans|244|PF00150.13[10-243]|noSP
GH5_2[nematode]|AER27772.1|Pratylenchus penetrans|195|PF00150.13[1-194]|noSP
GH5_2[nematode]|AER27764.1|Pratylenchus convallariae|246|PF00150.13[11-245]|noSP
GH5_2[nematode]|AER27771.1|Pratylenchus penetrans|195|PF00150.13[1-194]|noSP
GH5_2[nematode]|AER27776.1|Pratylenchus pratensis|85|PF00150.13[9-85]|noSP
GH5_2[nematode]|AER27779.1|Pratylenchus pratensis|163|PF00150.13[9-161]|noSP
GH5_2[nematode]|AER27790.1|Pratylenchus vulnus|242|PF00150.13[8-241]|noSP
GH5_2[nematode]|BAB68523.1|Pratylenchus penetrans|328|PF00150.13[38-284]|SP[1-21]
GH5_2[nematode]|AER27769.1|Pratylenchus neglectus|245|PF00150.13[10-245]|noSP
GH5_2[nematode]|AER27770.1|Pratylenchus neglectus|244|PF00150.13[10-243]|noSP
GH5_2[nematode]|AER27768.1|Pratylenchus neglectus|150|PF00150.13[2-149]|noSP
GH5_2[nematode]|AER27793.1|Hirschmanniella loofi|85|PF00150.13[8-85]|noSP
GH5_2[nematode]|Fgk|Aphelenchoides besseyi|548|PF00150.13[258-503]|SP[1-20]
GH5_2[nematode]|Fsx|Aphelenchoides besseyi|548|PF00150.13[258-503]|SP[1-20]
GH5_2[nematode]|Fm|Aphelenchoides besseyi|548|PF00150.13[258-503]|SP[1-20]
GH5_2[nematode]|ABY52965.1|Ditylenchus africanus|464|PF00150.13[36-277] PF00553.14[369-457]|SP[1-18]
GH5_2[nematode]|ACJ60676.1|Ditylenchus destructor|481|PF00150.13[36-275] PF00553.14[385-460]|SP[1-18]
GH5_2[nematode]|ACP20205.1|Ditylenchus destructor|338|PF00150.13[34-274]|SP[1-16]
GH5_2[nematode]|AFD33558.1|Aphelenchoides fragariae|320|PF00150.13[34-278]|SP[1-17]
GH5_2[nematode]|AFI63769.1|Aphelenchoides fragariae|236|PF00150.13[37-233]|SP[1-19]
GH5_2[nematode]|AER27797.1|Hirschmanniella gracilis|242|PF00150.13[8-241]|noSP
GH5_2[nematode]|AER27799.1|Hirschmanniella gracilis|242|PF00150.13[8-241]|noSP
GH5_2[nematode]|AER27794.1|Hirschmanniella loofi|162|PF00150.13[8-161]|noSP
GH5_2[nematode]|AER27795.1|Hirschmanniella loofi|153|PF00150.13[2-152]|noSP
GH5_2[nematode]|AER27798.1|Hirschmanniella gracilis|242|PF00150.13[9-238]|noSP
GH5_2[nematode]|AER27796.1|Hirschmanniella loofi|143|PF00150.13[5-139]|noSP
GH5_2[nematode]|AER27787.1|Pratylenchus vulnus|149|PF00150.13[4-145]|noSP
GH5_2[nematode]|AER27788.1|Pratylenchus vulnus|104|PF00150.13[3-93]|noSP
GH5_2[nematode]|AER27765.1|Pratylenchus crenatus|149|PF00150.13[4-145]|noSP
GH5_2[nematode]|AER27773.1|Pratylenchus penetrans|161|PF00150.13[10-160]|noSP
GH5_2[nematode]|AER27766.1|Pratylenchus crenatus|241|PF00150.13[11-237]|noSP
GH5_2[nematode]|AER27767.1|Pratylenchus crenatus|240|PF00150.13[11-236]|noSP
GH5_2[nematode]|ACB38289.1|Radopholus similis|467|PF00150.13[42-283] PF00553.14[374-458]|SP[1-22]
GH5_2[nematode]|GPLIN_000616300|Globodera pallida|450|PF00150.13[40-282] PF00553.14[356-443]|SP[1-19]
GH5_2[nematode]|GPLIN_000694900|Globodera pallida|425|PF00150.13[40-233] PF00553.14[331-418]|SP[1-19]
GH5_2[nematode]|AAK85303.1|Heterodera glycines|352|PF00150.13[35-278]|SP[1-18]
GH5_2[nematode]|AAC33848.1|Heterodera glycines|319|PF00150.13[35-278]|SP[1-18]
GH5_2[nematode]|CAC12959.1|Heterodera schachtii|365|PF00150.13[35-277]|SP[1-18]
GH5_2[nematode]|ACO55952.1|Heterodera avenae|463|PF00150.13[44-286] PF00553.14[374-455]|SP[1-22]
GH5_2[nematode]|AFQ55680.1|Heterodera avenae|333|PF00150.13[39-288]|SP[1-20]
GH5_2[nematode]|AAC48327.1|Heterodera glycines|476|PF00150.13[46-289] PF00553.14[386-463]|SP[1-22]
GH5_2[nematode]|CAC12958.1|Heterodera schachtii|418|PF00150.13[14-232] PF00553.14[328-405]|noSP
GH5_2[nematode]|AAC48325.1|Globodera rostochiensis|470|PF00150.13[35-281] PF00553.14[375-458]|SP[1-16]
GH5_2[nematode]|AAD56392.1|Globodera tabacum|470|PF00150.13[36-281] PF00553.14[375-458]|SP[1-16]
GH5_2[nematode]|GPLIN_000536400|Globodera pallida|365|PF00150.13[14-160] PF00553.14[270-361]|noSP
GH5_2[nematode]|AAC48341.1|Globodera rostochiensis|392|PF00150.13[44-289]|SP[1-22]
GH5_2[nematode]|AAD56393.1|Globodera tabacum|395|PF00150.13[44-289]|SP[1-24]
GH5_2[nematode]|GPLIN_000755100|Globodera pallida|312|PF00150.13[44-194]|SP[1-23]
GH5_2[nematode]|GPLIN_000755200|Globodera pallida|315|PF00150.13[44-272]|SP[1-23]
GH5_2[nematode]|ABV54448.1|Radopholus similis|319|PF00150.13[35-277]|SP[1-16]
GH5_2[nematode]|ABV54449.1|Radopholus similis|343|PF00150.13[43-282]|SP[1-22]
GH5_2[nematode]|AAN03646.1|Globodera rostochiensis|336|PF00150.13[45-290]|SP[1-19]
GH5_2[nematode]|AAN03647.1|Globodera rostochiensis|336|PF00150.13[45-290]|SP[1-27]
GH5_2[nematode]|GPLIN_001111200|Globodera pallida|336|PF00150.13[46-290]|SP[1-18]
GH5_2[nematode]|AAN32884.1|Heterodera glycines|335|PF00150.13[48-288]|SP[1-27]
GH5_2[nematode]|GPLIN_000779200|Globodera pallida|730|PF00150.13[318-561] PF00553.14[634-729]|SP[1-23]
GH5_2[nematode]|ADM72857.1|Rotylenchulus reniformis|414|PF00150.13[127-371]|SP[1-23]
GH5_2[nematode]|GPLIN_000304900|Globodera pallida|670|PF00150.13[390-623]|noSP
GH5_2[nematode]|GPLIN_000827200|Globodera pallida|307|PF00150.13[91-260]|SP[1-27]
GH5_2[nematode]|AJD14760.1|Pratylenchus goodeyi|341|PF00150.13[42-292]|SP[1-20]
GH5_2[nematode]|AER27775.1|Pratylenchus pratensis|197|PF00150.13[8-196]|noSP
GH5_2[nematode]|Minc13502|Meloidogyne incognita|281|PF00150.13[2-238]|noSP
GH5_2[nematode]|Minc19126|Meloidogyne incognita|278|PF00150.13[61-275]|noSP
GH5_2[nematode]|GPLIN_000313600|Globodera pallida|335|PF00150.13[40-290]|SP[1-22]
GH5_2[nematode]|GPLIN_001185800|Globodera pallida|562|PF00150.13[268-518]|SP[1-20]
GH5_2[nematode]|MhA1_Contig188.frz3.gene1|Meloidogyne hapla|294|PF00150.13[41-228]|SP[1-23]
GH5_2[nematode]|Minc18711|Meloidogyne incognita|331|PF00150.13[37-289]|SP[1-19]
GH5_2[nematode]|AER27807.1|Meloidogyne chitwoodi|294|PF00150.13[41-292]|SP[1-22]
GH5_2[nematode]|MhA1_Contig185.frz3.gene18|Meloidogyne hapla|274|PF00150.13[46-259]|noSP
GH5_2[nematode]|Minc10405|Meloidogyne incognita|322|PF00150.13[37-279]|SP[1-20]
GH5_2[nematode]|Minc18697|Meloidogyne incognita|258|PF00150.13[48-252]|SP[1-23]
GH5_2[nematode]|Minc19090|Meloidogyne incognita|304|PF00150.13[48-252]|SP[1-23]
GH5_2[nematode]|Minc18311|Meloidogyne incognita|220|PF00150.13[1-168]|noSP
GH5_2[nematode]|MhA1_Contig802.frz3.gene9|Meloidogyne hapla|335|PF00150.13[42-294]|SP[1-25]
GH5_2[nematode]|Minc13614|Meloidogyne incognita|336|PF00150.13[42-295]|SP[1-26]
GH5_2[nematode]|AER27784.1|Pratylenchus vulnus|204|PF00150.13[1-136]|noSP
GH5_2[nematode]|ADY92762.1|Koerneria sudhausi|207|PF00150.13[2-198]|noSP
GH5_2[nematode]|BAI44493.1|Aphelenchus avenae|327|PF00150.13[39-285]|SP[1-19]
GH5_2[eukaryotic]|BAB86867.1|Psacothea hilaris|325|PF00150.13[43-289]|SP[1-21]
GH5_2[eukaryotic]|AAX18655.1|Apriona germari|325|PF00150.13[43-289]|SP[1-13]
GH5_2[eukaryotic]|AHI15751.1|Apriona japonica|325|PF00150.13[43-289]|SP[1-21]
GH5_2[eukaryotic]|AHI15749.1|Apriona japonica|324|PF00150.13[43-289]|SP[1-21]
GH5_2[eukaryotic]|AHI15750.1|Apriona japonica|325|PF00150.13[44-290]|SP[1-22]
GH5_2[eukaryotic]|AHI15747.1|Apriona japonica|322|PF00150.13[41-287]|SP[1-19]
GH5_2[eukaryotic]|AFN89566.1|Anoplophora chinensis|325|PF00150.13[44-287]|SP[1-22]
GH5_2[eukaryotic]|AHI15748.1|Apriona japonica|323|PF00150.13[42-285]|SP[1-20]
GH5_2[eukaryotic]|ADI24131.1|Oncideres albomarginata chamela|323|PF00150.13[43-288]|SP[1-21]
GH5_2[eukaryotic]|AHI15746.1|Apriona japonica|320|PF00150.13[41-285]|SP[1-19]
GH5_2[prokaryotic]|AGA77897.1|Echinicola vietnamensis DSM 17526|323|PF00150.13[44-286]|SP[1-21]
GH5_2[prokaryotic]|CBK67187.1|Bacteroides xylanisolvens XB1A|327|PF00150.13[41-282]|SP[1-22]
GH5_2[eukaryotic]|BAD90558.1|Spirotrichonympha leidyi|307|PF00150.13[30-270]|SP[1-18]
GH5_2[prokaryotic]|ADO56545.1|Paenibacillus polymyxa SC2|493|PF00150.13[61-299]|SP[1-27]
GH5_2[prokaryotic]|AGK81015.1|Streptomyces fulvissimus DSM 40593|361|PF00150.13[74-324]|noSP
GH5_2[prokaryotic]|CAM03275.1|Saccharopolyspora erythraea NRRL 2338|335|PF00150.13[52-299]|SP[1-25]
GH5_2[prokaryotic]|AAZ54939.1|Thermobifida fusca YX|466|PF00553.14[40-136] PF00150.13[179-429]|SP[1-36]
GH5_2[prokaryotic]|AKN70681.1|Streptomyces sp. PBH53|463|PF00553.14[37-137] PF00150.13[169-419]|SP[1-16]
GH5_2[prokaryotic]|ACR74405.1|Eubacterium rectale ATCC 33656|388|PF00150.13[95-344]|SP[1-16]
GH5_2[prokaryotic]|CUH91792.1|Herbinix sp. SD1D|473|PF00150.13[51-297] PF00553.14[372-470]|SP[1-36]
GH5_26[prokaryotic]|AHM60064.1|Flammeovirgaceae bacterium 311|327|PF00150.13[29-294]|noSP
GH5_26[prokaryotic]|CCG99506.1|Fibrella aestuarina BUZ 2|337|PF00150.13[40-305]|SP[1-21]
GH5_26[prokaryotic]|AKD02203.1|Pontibacter korlensis|338|PF00150.13[43-308]|SP[1-23]
GH5_26[prokaryotic]|AEI50006.1|Runella slithyformis DSM 19594|337|PF00150.13[40-304]|SP[1-22]
GH5_26[prokaryotic]|ACR12128.1|Teredinibacter turnerae T7901|368|PF00150.13[67-338]|SP[1-27]
GH5_26[prokaryotic]|AEN71978.1|Rhodothermus marinus SG0.5JP17-172|357|PF00150.13[89-324]|SP[1-16]
GH5_26[prokaryotic]|AFN73747.1|Melioribacter roseus P3M-2|352|PF00150.13[59-322]|SP[1-22]
GH5_26[prokaryotic]|ACB74377.1|Opitutus terrae PB90-1|566|PF00150.13[198-439]|SP[1-23]
GH5_1[prokaryotic]|ADJ46955.1|Amycolatopsis mediterranei U32|509|PF00150.13[48-363] PF00553.14[408-507]|SP[1-25]
GH5_1[prokaryotic]|AGL16994.1|Actinoplanes sp. N902-109|533|PF00150.13[53-379] PF00553.14[434-532]|SP[1-27]
GH5_1[prokaryotic]|ADO57917.1|Paenibacillus polymyxa SC2|397|PF00150.13[47-356]|noSP
GH5_1[prokaryotic]|AFY33503.1|Calothrix sp. PCC 7507|527|PF00150.13[64-374] PF00553.14[427-511]|noSP
GH5_1[prokaryotic]|AEJ62365.1|Spirochaeta thermophila DSM 6578|528|PF00150.13[48-363]|SP[1-21]
GH5_1[nematode]|WBGene00102782|Pristionchus pacificus|325|PF00150.13[11-127]|noSP
GH5_1[nematode]|WBGene00117158|Pristionchus pacificus|370|PF00150.13[43-316]|SP[1-16]
0.4

S5 Fig. Full phylogeny of GH5 orthologues. Fields in taxa names include: subfamily of GH5, protein sequence id, specie, sequence length (aa), pfam domain composition, and signal peptide information. Part of the taxa were colored by the rules proposed by Katarzyna Rybarczyk-Mydłowska et al. [1]. For gene structure of our *Aphelenchoides besseyi Abe GH5-1* gene, please refer to Fig 6.

Reference:

1. **Rybarczyk-Mydłowska K, Maboreke HR, van Megen H, van den Elsen S, Mooyman P, Smant G, et al. Rather than by direct acquisition via lateral gene transfer, GHF5 cellulases were passed on from early Pratylenchidae to root-knot and cyst nematodes. BMC evolutionary biology. 2012;12(1):221.**
