# Supplementary material for: Glycoside Hydrolase (GH) 45 and 5 Candidate Cellulases in Aphelenchoides besseyi Isolated from Bird’s-Nest Fern
Source: PLoS One. 2016 Jul 8;11(7):e0158663. doi: 10.1371/journal.pone.0158663 (PMC4938546; doi:10.1371/journal.pone.0158663)
Supplement: S6 Fig — (PDF) [file pone.0158663.s006.pdf]

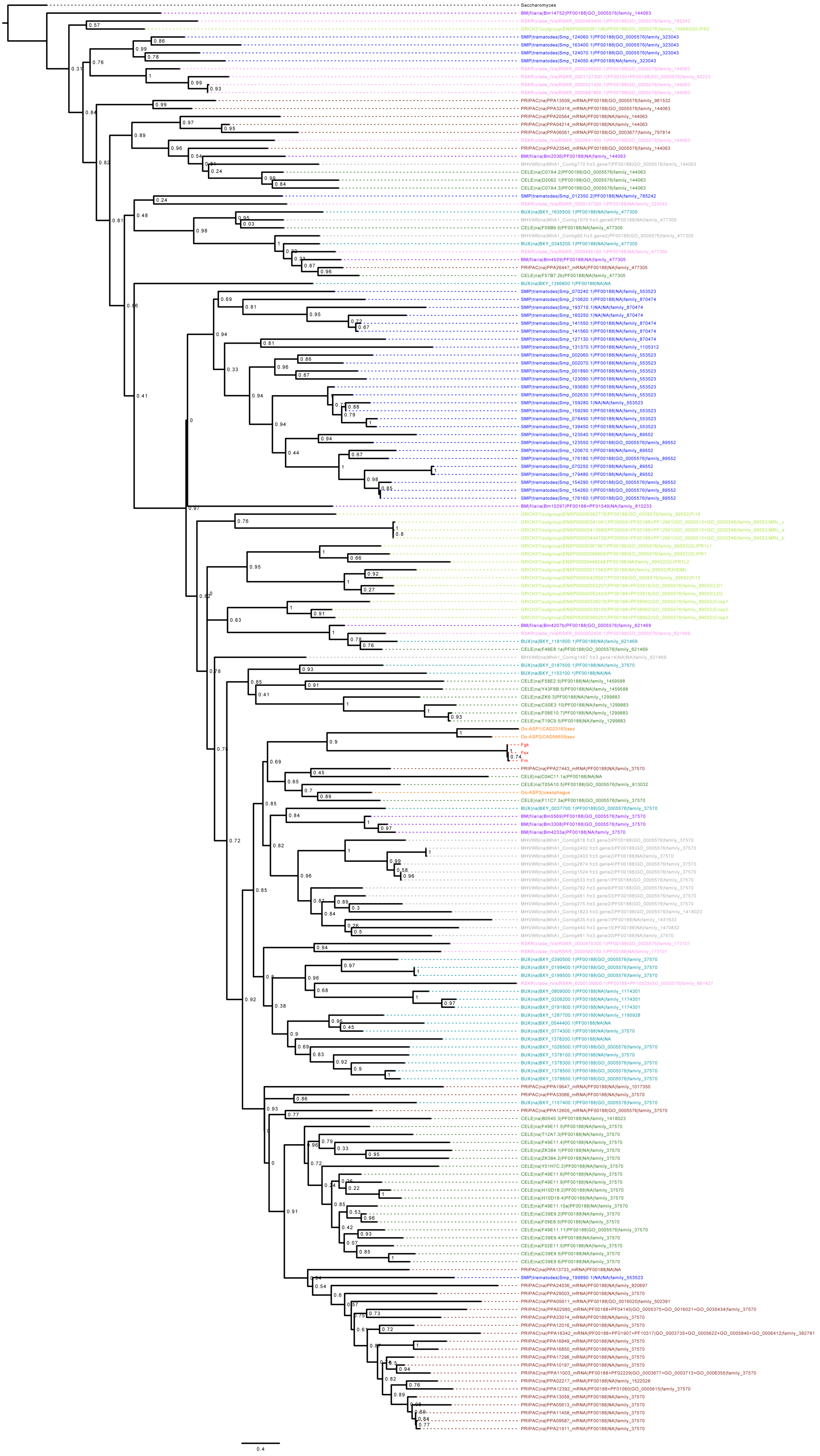
Saccharomyces
0.4

S6 Fig. A maximum likelihood phylogeny of SCP proteins. Our sequence of *Aphelenchoides besseyi* are labeled in red, and those of *Ostertagia ostertagi*, including Oo-ASP1 to Oo-ASP3, are labeled in orange. The phylogeny shows that our *A. besseyi* GH5-1 proteins and the orthologues expressed in reproductive organs of *O. ostertagi* are clustered with a high bootstrap value (0.9). The other species in this phylogeny include PRIPAC (*Pristionchus pacificus*), BM (Brugia malayi), RSKR (*Rhabditophanes* sp. kr3021), MHVW9 (*Meloidogyne hapla*), BUX (*Bursaphelenchus xylophilus*), GRCH37 (*Homo sapiens*), CELE (*Caenorhabditis elegans*), and SMP (*Schistosoma mansoni*). The name fields include specie, family, sequence id, pfam domain, gene ontology terms, and protein classification. The sequences were aligned using MAFFT (v7.271, options –localpair –maxiterate 1,000 –bl 30 [1]). The alignment was then trimmed with TrimAL (v1.2, options –gt0.12 –w 5 [2]). The phylogeny was then computed using FastTree (version 2.1.7 SSE3 [3]) with models of Wag and GAMMA

Reference:

1. Katoh K, Standley DM. MAFFT multiple sequence alignment software version 7: improvements in performance and usability. Mol Biol Evol. 2013;30(4):772-80. doi: 10.1093/molbev/mst010. PubMed PMID: 23329690; PubMed Central PMCID: PMCPMC3603318.

2. Capella-Gutierrez S, Silla-Martinez JM, Gabaldon T. trimAl: a tool for automated alignment trimming in large-scale phylogenetic analyses. Bioinformatics. 2009;25(15):1972-3. doi: 10.1093/bioinformatics/btp348. PubMed PMID: 19505945; PubMed Central PMCID: PMCPMC2712344.

3. Price MN, Dehal PS, Arkin AP. FastTree 2–approximately maximum-likelihood trees for large alignments. PloS one. 2010;5(3):e9490.
