# Supplementary material for: Glycoside Hydrolase (GH) 45 and 5 Candidate Cellulases in Aphelenchoides besseyi Isolated from Bird’s-Nest Fern
Source: PLoS One. 2016 Jul 8;11(7):e0158663. doi: 10.1371/journal.pone.0158663 (PMC4938546; doi:10.1371/journal.pone.0158663)
Supplement: S1 Table — (PDF) [file pone.0158663.s007.pdf]

S1 Table. **Primers used to amplify *Aphelenchoides besseyi* cDNA and genomic DNA*.**

Primer	Sequence (5' → 3')	Description
988F	CTCAAAGATTAAGCCATGC	18S [1]
1912R	TTTACGGTCAGAACTAGGG	
M-1813F	CTGCGCGAGAGGTGAAAT	18S (modified from [1])
M-2646R	ACCTACTGATACCTTGTTACGACTTTT	
ENG-1	TAYGTIATHGTIGAYTGGCA	Fragment amplification
ENG-2	GTICCRTAYTCIGTIACRAA	
GH45 ENG-1	ACIMGITAYTGGGAYTGYTG	Fragment amplification
GH45 ENG-2	RCAICCRTTRAAIADICCIAC	
Abe GH5-1-F	TGAAGTGAACTTTGGCAAGAAATGAAAATC	cDNA amplification
Abe GH5-1-R	GACTGATCATTTTAACGAGCTTAACAACTG	
Abe GH45-2-F	CCAACAAAATGGTTCAATTCATT	cDNA amplification
Abe GH45-2-R	CGTTTCAACAATGACATTGTACTCTT	
Abe GH45-3-F	CCAACAAAAATGGTTCAATTCATC	cDNA amplification
Abe GH45-3-R	GTTATTAACTGTCTGCACGAA	
FmGH5i-F	TCGCAATACAATCCTCAGAAG	Inverse PCR
FmGH5i-R	GAGTTTAAGCGTCGATAGAAGATG	
AbeFm-GH5-GSP-F	TACGTGATTGTTGATTGGCACGGAA	3'RACE
AbeFm-GH5-NGSP-F	CGATACCCCAATTGAAAGTCCGATT	
AbeFm-GH5-GSP-R	CCATTGGCGCATAGCATTGTAAA	5'RACE
AbeFm-GH45-GSP-F	TATTGGGACTGCTGCAAACCATCTT	3'RACE
AbeFm-GH45-GSP-R	TCCGCCGGTATTGGTAACTTGAAC	5'RACE
AbeRl-GH45-GSP-F	GCGGTATTGGGACTGTTGCAAG	3'RACE
AbeRl-GH45-GSP-R	GCAGCCGTTGAACAAGCC	5'RACE

AUAP	GGCCACGCGTCGACTAGTAC	3’RACE and 5’RACE
Abe GH5-1-S-F	ATGCGAACCTCAGTGGGGTTT	Southern blot
Abe GH5-1-S-R	GTTCCGTATTCGGTTACAAATAAAG	
Abe GH5-1-ISH-F	TTCCACAATATGATGATACTACTCCAA	*In situ* hybridization
Abe GH5-1-ISH-R	GATCATTTTAACGAGCTTAACAACTG	
T7- Abe GH5-1-ISH-F	TAATACGACTCACTATAGGGTTCCACAATATGATGATACTACTCCAA	
T7- Abe GH5-1-ISH-R	TAATACGACTCACTATAGGGGATCATTTTAACGAGCTTAACAACTG	

*The T7-promoter sequence to prepare DNA template for *in vitro* transcription is underlined.

Refernece:

1. Holterman M, van der Wurff A, van den Elsen S, van Megen H, Bongers T, Holovachov O, et al. Phylum-wide analysis of SSU rDNA reveals deep phylogenetic relationships among nematodes and accelerated evolution toward crown Clades. Mol Biol Evol. 2006;23(9):1792-800. doi: 10.1093/molbev/msl044. PubMed PMID: 16790472.
